# Integrative systems biology analysis of barley transcriptome ─ hormonal signaling against biotic stress

**DOI:** 10.1371/journal.pone.0281470

**Published:** 2023-04-27

**Authors:** Zahra Soltani, Ali Moghadam, Ahmad Tahmasebi, Ali Niazi

**Affiliations:** Institute of Biotechnology, Shiraz University, Shiraz, Iran; Universidade de Lisboa Instituto Superior de Agronomia, PORTUGAL

## Abstract

Biotic stresses are pests and pathogens that cause a variety of crop diseases and damages. In response to these agents, crops trigger specific defense signal transduction pathways in which hormones play a central role. To recognize hormonal signaling, we integrated barley transcriptome datasets related to hormonal treatments and biotic stresses. In the meta-analysis of each dataset, 308 hormonal and 1232 biotic DEGs were identified respectively. According to the results, 24 biotic TFs belonging to 15 conserved families and 6 hormonal TFs belonging to 6 conserved families were identified, with the NF-YC, GNAT, and WHIRLY families being the most prevalent. Additionally, gene enrichment and pathway analyses revealed that over-represented cis-acting elements were recognized in response to pathogens and hormones. Based on the co-expression analysis, 6 biotic and 7 hormonal modules were uncovered. Finally, the hub genes of *PKT3*, *PR1*, *SSI2*, *LOX2*, *OPR3*, and *AOS* were candidates for further study in JA- or SA-mediated plant defense. The qPCR confirmed that the expression of these genes was induced from 3 to 6 h following exposure to 100 μM MeJA, with peak expression occurring between 12 h and 24 h and decreasing after 48 h. Overexpression of *PR1* was one of the first steps toward SAR. As well as regulating SAR, *NPR1* has also been shown to be involved in the activation of ISR by the *SSI2*. *LOX2* catalyzes the first step of JA biosynthesis, *PKT3* plays an important role in wound-activated responses, and *OPR3* and *AOS* are involved in JA biosynthesis. In addition, many unknown genes were introduced that can be used by crop biotechnologists to accelerate barley genetic engineering.

## Introduction

Cereals are directly related to the food source and energy supply and are always fronted with a wide range of biotic stresses that negatively affect their survival and growth [1, 2]. These stresses can cause by living organisms such as bacteria, viruses, fungi, nematodes, and pests [1, 3]. Among biotic stresses, fungal pathogens play a fundamental role in causing 70 to 80% of cereal diseases [4, 5]. Barley (*Hordeum vulgar*) is very susceptible to fungal pathogens [6] like *Fusarium graminearum* and *Puccinia hordei*, which are the serious threats in wet or semi-wet regions [7, 8]. On the other hand, pests are other destructive pathogens of cereals, which reduce plant growth without specific leaf symptoms [9]. Hence, identification of the key genes related to the resistance is the first step in development of new breeding lines to manage the diseases [10–12]. For example, the breeding for barley resistance to *Rhopalosiphum padi* has revealed that several resistance genes are associated with the decrease in aphid growth [13]. Several gain-of-function and loss-of-function mutants have shown the genes related to fundamental defense and effector-triggered immunity [14].

Today, large-scale data generated from functional genomic studies on plant-pathogen interactions have provided massive information [15]. Therefore, access to OMICS information on the behavior of defense genes against pathogens helps to decipher the gene networks that led to the identification of pathogen specific-responses [16]. In recent years, computational systems biology has collected exquisite opportunities to dominate biological complexity [17, 18]. Systems biology approaches, including meta-analysis and gene network analysis, are well-known strategies for integrating these data and discovering particular molecular interactions [19, 20]. These approaches provide a robust statistical framework for re-evaluating key findings, improving sensitivity by increasing sample size, testing new hypotheses, and characterizing novel gene candidates for plant breeding programs [21–24].

Therefore, progress in whole-genome transcriptome analysis allows the analysis of gene regulation and plant-pathogen interactions in various conditions, allowing the identification of pathogen-specific biomolecular networks and hormonal signaling pathways. For example, the important responses to pathogens may be due to the cross-talks among hormone-related pathways [25] such as ethylene (ET), salicylic acid (SA), jasmonic acid (JA), abscisic acid (ABA), and/or signaling due to calcium [26], activated oxygen species (ROS) [27], and phosphorylation chains [28]. Although many signaling pathways interact, regulatory factors such as transcription factors (TFs), microRNAs (miRNAs), and protein kinases (PKs) regulate plant defense responses. Most plant immune responses are transcriptionally controlled [29, 30] and some pathogen-derived factors disrupt host transcriptional complexes to increase their virulence, highlighting the importance of transcriptional regulation of host defenses [31].

In defense responses, TFs play distinct roles in modulating gene expression and activating signaling cascades [32, 33]. It has shown that the WRKY family, which is considered the core of the plant immune system, plays an essential role in phytohormone signaling and pathogen defense [34]. Members of MYB/bHLH regulate distinct cellular processes, such as responses to biotic stresses, hormonal signaling, and hypersensitive reaction (HR). In addition, the bZIP family plays an important role in regulating pathogen-responsive pathways and mitigating pathogens [35].

Plants are equipped with an array of defense strategies against pathogens [36]. Systemic acquired resistance (SAR) and induced systemic resistance (ISR) are two forms of induced resistance pathways [37]. ISR is a nonspecific resistance mechanism that is activated by infection, and acts against a considerably broader spectrum of both (hemi-) biotrophic and necrotrophic pathogens [37, 38]. On the other hand, SAR in systemic and uninfected tissues provides long-term protection against (hemi-) biotrophic pathogens. In addition, SAR is mostly studied as a leaf-to-leaf response [39] and is activated during the local defense response [39], while ISR is triggered by the colonization of roots by certain non-pathogenic rhizosphere microorganisms such as plant growth-promoting rhizobacteria (PGPR) and plant growth-promoting fungi [37–40].

Many hormonal signaling are involved in both defense systems. For example, SAR requires the synthesis of SA, which triggers the expression of a well-known set of pathogen-related (*PRs*) genes [41, 42], while ISR is dependent on JA and ET signaling pathways [43]. These accumulating signaling molecules moderate the defense responses, and when they are used exogenously, they are enough to induce the resistance [39]. However, ISR and SAR together present a better protection than each of them alone, and this shows that they can act increasingly in inducing resistance to the pathogens [39]. Furthermore, plants rarely encounter only one stressor in a natural habitat when they evaluate defense responses. Therefore, understanding the interactions between SAR/ISR and other signaling cascades help to raise our knowledge for strengthening the plant defense against biotic stresses.

Many studies on SA-mediated plant defense have demonstrated that SA is not only a major regulator of SAR and induces the *PRs*, but also this hormone is necessary to induce the HR [44]. There is a complex network of SA signaling mediated by the NPR1 and mitogen-activated protein kinases (MAPKs) [45]. The HR control by TFs such as MYB depends on SA accumulation, but not on NPR1, a signaling protein downstream of SA accumulation [46]. Moreover, SA-dependent defense responses are effective against biotrophic pathogens that auto-activate the HR [46]. Further, it has shown that the suppression of *SSI2* reduces the 18:1 fatty acids and induces phenylalanine ammonia-lyase-mediated SA accumulation, which auto-activates the HR [47].

To gain a clear insight into the mechanisms involved in the response of barley to various pathogens and hormonal signaling, we used meta-analysis and co-expression gene network analysis of barley transcriptome. We identified some key genes and significant gene networks in response to pathogens and hormones. These genes were identified through gene enrichment analysis of metabolite pathways, TFs, PKs, and microRNAs.

For instance, TFs interact with enhancers to coordinate gene expression transcriptionally [48], while miRNAs are gene regulators transcriptionally or post-transcriptionally [49]. Since both regulators demonstrate great impact on plant genetic systems, the circuiting of miRNAs-TFs will allow the orchestration of diverse biological processes with high reliability [49, 50]. Therefore, observing and understanding a dynamic relationship among these regulators is interesting. In addition, some significant hormone- and pathogen-responsive hub genes were validated after methyl jasmonic acid (MeJA) treatment. Plant protection could be facilitated by the exogenous application of the MeJA, which has demonstrated great potential as a defense inducer for fungi and pest attacks [51]. The MeJA stimulates induced systemic SA/JA/ET pathways in the infected tissues, thus inducing the expression of genes related to the resistance. MeJA affects many genes related to both SAR and ISR pathways. Previous studies have shown that *PR1*, *AOS*, and *LOX2* respond positively to the MeJA treatment, protecting the plant from infection [52, 53]. Therefore, more accurate information on barley-pathogen interactions might be helpful for plant biotechnologists to supply the pathogen-tolerant cultivars.

## Materials and methods

**Fig 1:**
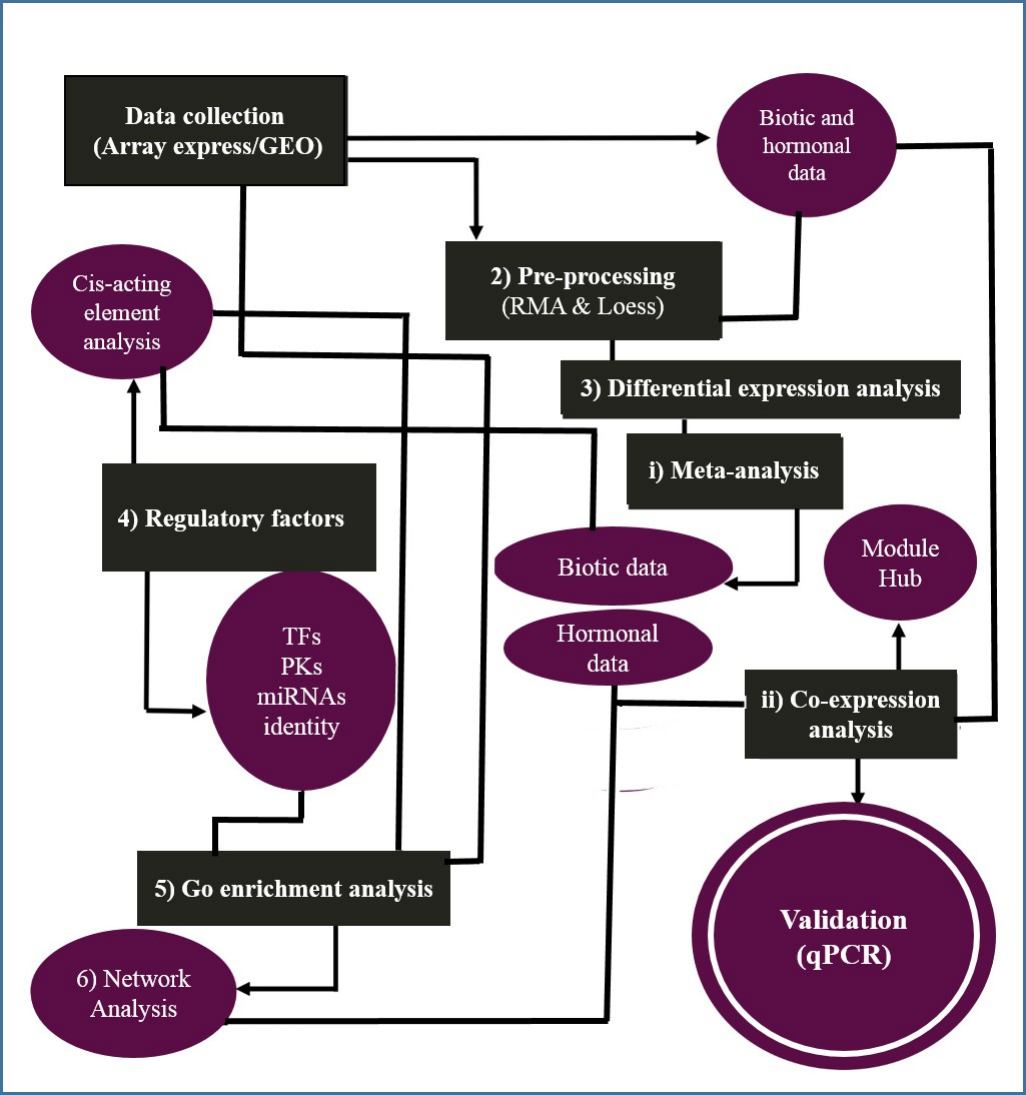
Schematic overview of the integrative strategy for understanding aspects of response of barley transcriptome to biotic and hormonal stresses.

We integrated the transcriptome datasets of barley through systems biology approaches to identify responsive key genes involved in the pathogen-hormone interactions. First, the meta-analysis discovered the DEGs, and then the co-expression gene network analysis grouped the DEGs into the modules (Fig 1).

### Data collection

The raw microarray expression data of barley exposed to diverse biotic stresses, including fungi, bacteria, viruses, and insects, or various hormonal treatments, including JA, gibberellic acid (GA), and MeJA, were retrieved from publicly available databases of Gene Expression Omnibus (GEO), Sequence Read Archive (SRA), and Array Express. The data selection was done based on the Minimal Information about a Microarray Experiment (MIAME) requirements [54]. Therefore, the studies with relatively similar genetic backgrounds without any mutated or transgenic samples were chosen with suitable quality and biological and technical replicates of controls and treatments [55]. The total data included ten studies with 479 biotic stress samples and four studies with 46 hormonal treatment samples (S1 Table). The raw data included two platforms, Affymetrix (Accession: GPL1340) and Agilent (Accessions: GPL14904 and GPL15513). The probe-gene maps and probe-annotation files were retrieved from the Affymetrix database.

### Pre-processing and normalization

The normalization of the Affymetrix data for each array was performed with Robust Multichip Average (RMA) algorithm [56] by the Expression Console software (V. 1.3.1). Briefly, after background correction of this platform by the RMA method, the Quantile method normalization was carried out, and then the Median Polish method was applied to summarize the probe sets [57]. Two color Agilent data were processed using Flex Array software (V. 1.6.3). Briefly, the background correction was performed using the Normexp method to estimate the intensity of expression values [58], and then the normalization was performed using the Quantile method [59]. For the normalization of one color Agilent data, we applied the LIMMA package and LOESS method in the R program [60].

### Meta-analysis and identification of DEGs

At the first, the control and treatment samples of each study were separately defined. Then the guardianship data, including TAIR IDs, was used for meta-analysis using the MetaDE R package. The merged datasets were filtered again except for 20% of the expressionless genes (with low expression intensity) and 20% of the non-educational genes (with quantitative changes). Finally, the RankProd method, a non-parametric method, was applied to genes rank based on the fold change (FC). Principally, the RankProd algorithm calculates pairwise FC with replicates for each gene between treatment and control samples in both directions, respectively, and converts FC into ranks among all genes, then searches for genes that are continuously top-ranked across replicates [61]. Converting FC into ranks overcomes the heterogeneity among multiple datasets and an overall ranked gene list is produced based on the *P-value* and the *False Discovery Rate* (*FDR*) of each gene. In this method, up- and down-regulated genes with log2 FC >1 or log2 FC <−1 and *FDR* ≤ 0.05 were considered significant differentially-expressed genes (DEGs) [62].

### Gene enrichment analyses

Gene ontology (GO) enrichment analysis of the DEGs to finding of the molecular pathways responsive to pathogens and hormones was performed using the Kyoto Encyclopedia of Genes and Genomes (KEGG) and DAVID resources [63, 64]. GO was performed on the unique list of TAIR IDs of the DEGs. The results were categorized in Molecular Function (MF), Biological Process (BP), and Cellular Component (CC). GO terms with the corrected *P-value* ≤ 0.05 were considered significant [65].

### Identification of TF, PK and miRNA families

The DEGs were BLASTed against the iTAK database [66] to identify the TFs and PKs. Next, the psRNATarget database [67] was used to identify candidate microRNAs. Then, the genes targeted by miRNAs were considered to GO analysis. The WEGO software [68] was used for plotting GO results, and the Agrigo software was used to enrich the GO terms.

### Gene co-expression network analysis and identification of modules

In order to identify the DEGs with similar expression pattern, a Weighted Gene Co-expression Network Analysis (WGCNA) in R space was performed on the matrix of normalized expression values. For this purpose, the meta-analysis output file (including DEGs) was merged with the initial normalized data and then the treatment and control samples were separated in the columns. First, a similarity matrix was calculated based on Pearson correlations between each DEG pair and converted into an adjacency matrix by applying the soft-thresholding β power. This power makes the adjacency matrix to be the continuous value between 0 and 1 and the co-expression similarity was raising to achieve scale-free topology. Following, the Topological Overlap Matrix (TOM) [69, 70] was calculated for hierarchical clustering analysis. Then, to discover modules, a dissimilarity matrix is obtained (dissTOM) that resulted in genes with similar expression clustered in the same gene module. Finally, a dynamic tree cut algorithm was used to identify co-expression gene modules. This method can detect nested clusters and have been shown to better detect outliers [71]. The major parameters were defined with a minimum size cut-off of 30 genes and a threshold of 0.3 and a minimum size cut-off of 100 genes and a threshold of 0.99 for avoiding abnormal modules in dendrogram, on the biotic and hormonal data respectively. As a result, an undirected weighted network with scale-free topology composed of modules of barley DEGs with correlated expression during pathogen infections was obtained.

### Identification of hub genes

Network visualization and calculation of topological properties for each module were performed using the Cytoscape software (V. 3.6.1). To identify the hub genes, computational algorithms of Maximal Clique Centrality (MCC) were applied using a plug-in of Cytoscape, CytoHubba [72, 73] followed by GO enrichment analysis by DAVID database.

### Promoter analysis

To find the upstream flanking region of DEGs (1000 bp) [74], a BLAST against the Ensemble plant database was performed [75]. Then, the MEME [76] database was used to identify the conserved cis-acting elements. The motifs were counted, and the abundance of regulatory elements in the promoter region of various genes was investigated. Next, we used Tomtom v 5.0.1 tool [77] to remove redundancy motifs and to determine known CRE based on the motif database of JASPAR (https://jaspar.genereg.net/) [78] with a threshold *E-value* of 0.05. GOMO tool [79] was also used to identify the biological roles of the cis-acting elements.

### Protein-protein interactions

The protein-protein interaction network (PPI) was constructed based on the both datasets (biotic stress/hormonal treatment). The unique list of TAIR IDs of each dataset was BLASTed against the STRING database with default parameters (with the highest required interaction score = 0.9). The highest confidence was selected to make more comprehensive network and to study more important connectivity’s. Finally, the network was drawn by Cytoscape software and key genes in networks were screened out by the score. In this network, high degree could represent essential genes [80]. In the network, nodes represent genes and edges represent the interactions between the nodes. The highly important nodes in the network, the hub nodes, were obtained according to the k-core.

### Plant materials and growth conditions

The seeds of *hordeum vulgare* genotype Oxin were sown in trays, germinated, and grown in a glasshouse in the pots that contained peat moss, perlite vermiculite, and soil (1:1:3) under a 16-h light:8-h dark cycle at 25°C (S1 Fig).

### Stress conditions and sampling

The seedlings in the 3 leaf-stage were sprayed and irrigated with 100 μM MeJA plus 0.1% Tween-20 in 0.1% ethanol. For the controls, 0.1% Tween-20 solution in 0.1% ethanol was used. Plastic covered all the pots. Leaf samples were collected at 3, 6, 24, and 48 hours after the treatment in three biological replicates. For RNA extraction, the leaves were immediately frozen in liquid nitrogen and stored at −80°C.

### RNA extraction, DNase treatment, and cDNA synthesis

Total RNA was extracted from leaf samples using a Column RNA Isolation Kit (DENAzist Asia Co., Mashhad Iran) according to the manufacturer’s instructions. The quantity and concentration of RNA were measured using a NanoDrop ND 1000 Spectrophotometer (Thermo Fisher Scientific, Wilmington, DE, USA). The integrity and quality of RNA were checked by visual observation of 28S and 18S rRNA bands on a 1.2% agarose gel. Before use, RNA samples were stored at −80°C. DNase treatment of RNA was carried out using the RNase-free DNase kit (Thermo Fisher Scientific, Waltham, Massachusetts, USA) according to the manufacturer’s instructions. The treated RNA was rechecked by NanoDrop and agarose gel. Then, 1 μg of DNase-treated RNA was used for first-strand cDNA synthesis using a SinaClon BioScience kit (Karaj, Iran) according to the manufacturer’s instructions. The cDNA samples were stored at −20°C prior to use.

### Selection of candidate genes for gene expression analysis

To validate the transcriptome data analysis, 6 genes with the highest interaction in the PPI network related to hormone signaling and disease resistance-related pathways were selected. These candidate genes are the most important hub genes that show interactions between SAR and ISR. These genes play a central role in defense responses and hormone signaling [81]. The first candidate gene is *SSI2* (*AT2G43710*), a hub gene in the biotic turquoise and brown modules, and hormonal blue module, which interacts with fatty acids (FAs) that affect defense signaling (S5 Fig). The *SSI2* represses plant defense by affecting the generation of a lipid molecule necessary for SA synthesis and the *NPR1*-independent pathway. Moreover, the SA and JA signaling-related regulatory genes inducing the *PRs* are activated in response to numerous pathogens and ultimately enhance resistance [82]. Among the *PRs*, *PR1* has been frequently used as a marker gene for SAR in many species [83]. As the second candidate gene, we selected *PR1* from the biotic brown module. The third is *PKT3* (*AT2G33150*) which is involved in long-chain fatty-acid beta-oxidation prior to gluconeogenesis during seed germination and seedling growth [84]. This enzyme was identified in the biotic brown and hormonal green modules that confers sensitivity to 2,4-dichlorophenoxybutiric acid (2,4-DB). Required on local and systemic induction of JA biosynthesis after wounding and pathogen infection and involved in JA biosynthesis during senescence [85].

Other candidate’s key genes, such as *AOS*, *OPR3*, and *LOX2*, showed a high positive correlation and interaction with each other. These genes were identified in biotic blue or hormonal turquoise modules, hormonal red modules, and biotic turquoise modules, respectively (S5 Fig). The induction of the JA-responsive marker gene by different JA derivatives was equally sensitive to SA-mediated suppression. Activation of genes encoding key enzymes in the JA biosynthesis pathway, such as LOX2, AOS, AOC2, and OPR3, was also repressed by SA, suggesting that the JA biosynthesis pathway may be a target for SA-mediated antagonism [81]. These show that JA biosynthesis is under the control of a positive feedback regulatory system and that the down-regulation of JA biosynthesis-responsive genes by SA may be a critical mechanism in SA–JA cross-talks [81].

### Primer design

Primers were designed using Allele ID 7 and Vector NTI 11 software for the reference and candidate genes (Table 1).

**Table 1:** Primer sequences used in the qPCR.

Gene	Accession number	Forward sequence	Reverse sequence	Ta (°C)	Product (bp)
*SSI2*	*AT2G43710*	TGGACTGCTGAGGAGAATAG	TTGGAAGGAGGTGTAGATGAA	62.5	171
*PR1*	*AT2G14610*	AGAAGGACTACGACTACG	TATGTATATGTACTGCGAAAAG	57	220
*PKT3*	*AT2G33150*	ATCGTCATCGTCGCTGCCTATA	CGCCAACTTCGCTCGGATT	67.5	142
*AOS*	*AT5G42650*	TAGCAGCAGAGTTAGATG	TGGAAGTAGTAGAAGTCG	57	137
*OPR3*	*AT2G06050*	GCGACTTCACACTCCATA	CCTTCTTCCAGCCTTCAA	60.5	222
*LOX2*	*AT3G45140*	GGACGACGAGATGAAGAAGT	GAGTGCGAGGAGAGGATG	63.3	117
*Actin*	*AT3G18780*	GGTGTTATGGTTGGTATGG	ATAGAAGGTGTGATGCCA	59.4	150

### Validation of candidate genes

First, primer specificity was confirmed by PCR and sequence analysis. To minimize pipetting error, the cDNA samples were diluted 1:20 by using nuclease-free water, and 5 μL cDNA was used for qPCR. Relative qPCR was performed in a 20 μL volume containing 5 μL cDNA (diluted), 10 μL RealQ Plus 2x Master Mix Green (Sinuhebiotech, Shiraz, Iran), and 0.7 μl of primers (10 μM). The amplification reactions were carried out in a Line-gene K thermal cycler (Bioer, China) under the following conditions: 15 sat 95°C, 45 cycles of 94°C for 15 s, Ta temperature for 15 s, and 72°C for 20 s. After 45 cycles, the specificity of the amplifications was tested by melting curve analysis by heating from 50 to 95°C. All amplification reactions were repeated three times under identical conditions and included a negative control. In addition, 6 serial dilution samples (1:10) provided using specific PCR products of a cDNA template for each primer were used in qPCR as positive controls. Besides, we used these standards to calculate the qPCR efficiency.

### qPCR data analysis

The relative expression was calculated according to the 2^─ΔΔCT^ method [86]. The 6 C_T_ values for each sample were calculated using the Line-gene K software. Three technical replicates were run for each sample in qPCR. The actin gene (*AT3G18780*) was used as the reference gene for data normalization [87, 88].

### Statistical analysis

Analysis of variance followed by Duncan’s multiple range test was performed using MINITAB (Minitab, Inc., Pennsylvania, USA). In all cases, differences were regarded to be statistically significant at a *P-value* ≤ 0.05. All experiments were performed in triplicate, illustrated using the GraphPad prism software (GraphPad Software, Inc., San Diego, CA).

## Results

### Pre-processing and normalization

Pre-processing was applied to the raw data to reduce the impact of existing noises. Since reducing heterogeneity among studies is a necessary step for the integration of the data, the normalization methods of RMA and LOESS were applied to Affymetrix and Agilent platforms, respectively. The proposed framework of the normalization reduced the batch effects and facilitated the direct merging (S2 Fig).

### Meta-analysis and identification of DEGs

In this study, the RankProd method was used to identify DEGs for biotic stress and hormonal treatment studies (S1 Table). The results of the meta-analysis may contain duplicated genes occurring by multiple probes mapped into the same gene symbols. These duplicated genes, if any, are filtered out by different methods after obtaining gene values for each dataset. For each pair of duplicated genes, the up or down-regulation was considered based on the smaller *P-value*. Thus, we found that from 44 duplicated genes in the biotic data, 10 genes remained in the form of up-regulated and 34 genes remained in the form of down-regulated, as well as from 4 duplicate genes in the hormonal data, 2 genes were considered as up-regulated and 2 genes considered as down-regulated.

**Fig 2:**
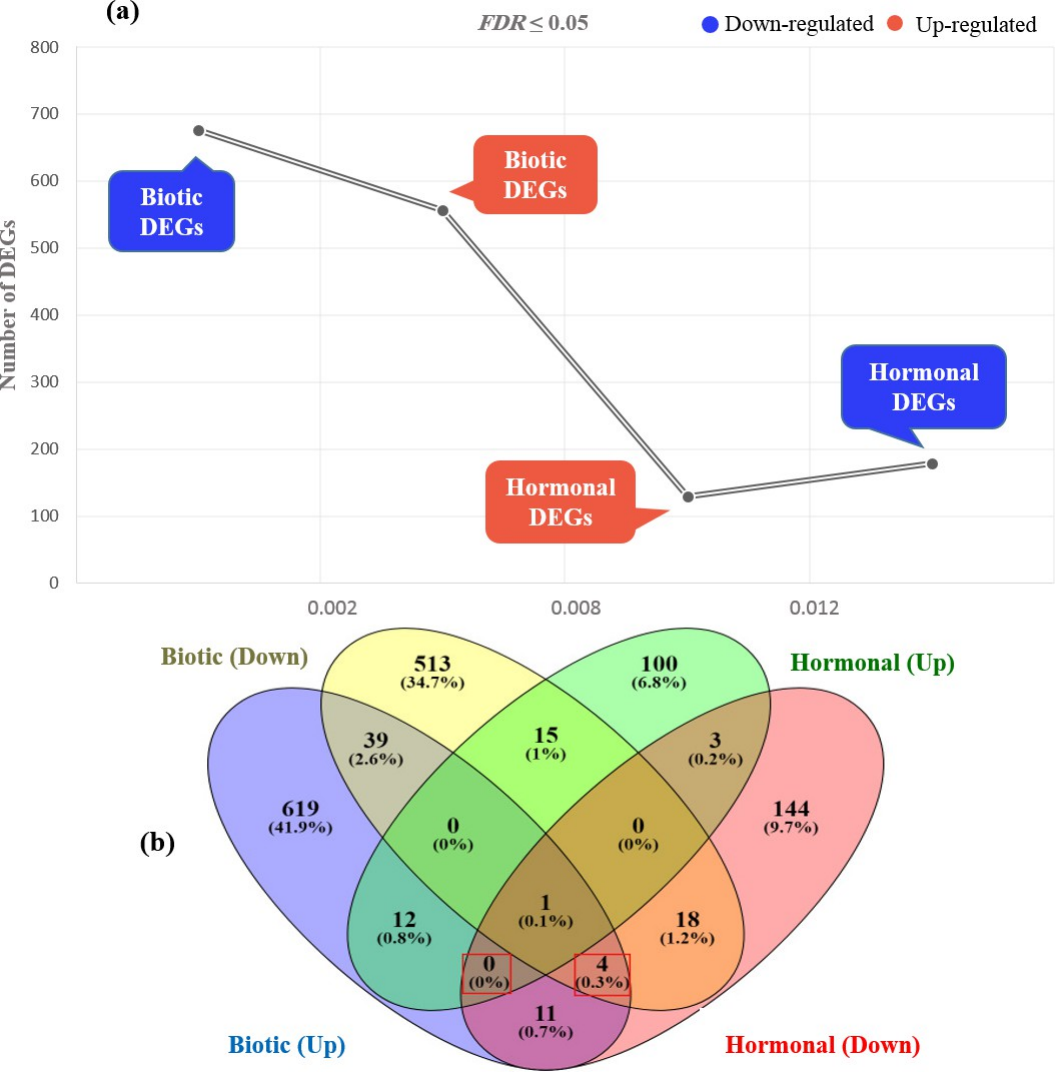
Venn diagrams illustrating the number of significant (*P-value* ≤ 0.05) down- and up-regulated DEGs between biotic and hormonal datasets. (a) For the biotic datasets, 556 up- and 676 down-regulated DEGs were identified. For the hormonal datasets, 179 up- and 129 down-regulated DEGs were identified. (b) 61 Common DEGs between the biotic and hormonal studies were illustrated. Four-way Venn diagrams show common and up-/down-regulated DEGs in response to biotic and hormonal stresses. In total, 12 common down-regulated DEGs and 18 common up-regulated DEGs were identified. The red rectangle highlights the total up- and down-regulated DEGs (*P-value* ≤ 0.05). In fact, the completely biotic DEGs are common to the up-regulated hormonal DEGs in the four DEGs, but there is no commonality between the whole hormonal DEGs and the down-regulated biotic DEGs.

Totally, 1540 DEGs were found, in which 1232 DEGs were from biotic datasets, and 308 DEGs were from hormonal datasets (*FDR* ≤ 0.05). In the biotic stresses, 556 DEGs were up-regulated and 676 DEGs were down-regulated, while in the hormonal treatments, 179 DEGs were up-regulated, and 129 DEGs were down-regulated (Fig 2 and S2 Table). Venn diagrams showed the numbers of specific and common DEGs between biotic and hormonal datasets (Fig 2).

Based on the results, 61 common DEGs between biotic and hormonal datasets were identified whereas 18 up-regulated DEGs such as *EMBRYO DEFECTIVE 1144* (*EMB1144*), *phenylalanine ammonia-lyase 1* (*PAL1*), *glutamic acid decarboxylase* (*GAD*), *aldehyde dehydrogenase family 7 member B4* (*ALDH7B4*), *jasmonate-Zim-domain protein 1* (*JAZ1*), and some genes with unknown function including *AT1G22410*, *AT1G06550*, *AT5G58950*, and *AT1G68300* were identified. In addition, a total of 12 down-regulated DEGs including *metallothionein 3* (*MT3*), *silent information regulator* (*SIR*), *ATP-binding cassette transporter-A2* (*ABCA2*), *vacuolar processing enzyme* (*GAMMA-VPE*), and *apurinic/apyrimidinic endodeoxyribonuclease 1* (*APE1*) as well as some genes with unknown function including *AT1G22410*, *AT3G15810*, *AT2G21180*, and *AT4G09890* were identified.

### Gene enrichment analysis of DEGs

**Fig 3:**
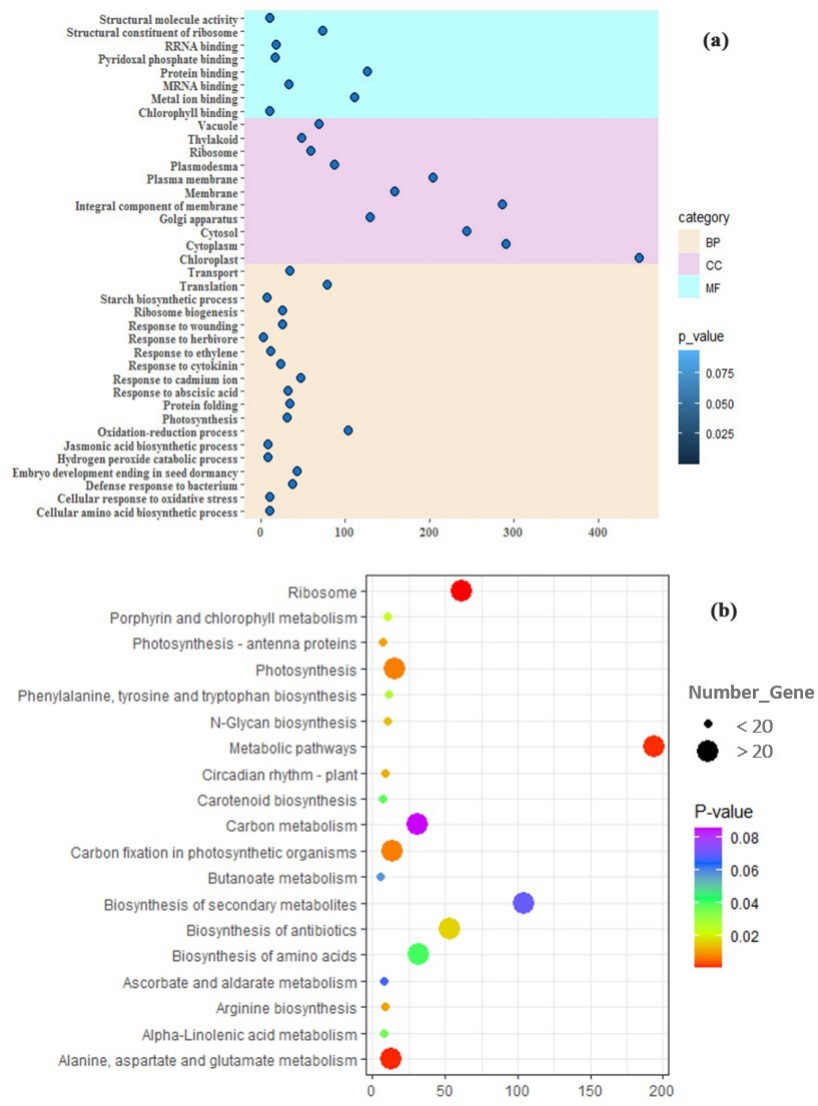
Gene enrichment and pathway analyses of biotic DEGs. (a) Gene ontology was performed using DAVID. The most significantly (*P-value* ≤ 0.05) enriched GO terms in Biological Process (BP), Molecular Function (MF), and Cellular Component (CC) categories related to the defense responses are presented. The horizontal axis shows the number of genes based on the *P-value*, while the vertical axis represents Go terms. (b) Shows KEGG pathway analysis of biotic DEGs. Y-axis shows the pathways and the X-axis shows the number of DEGs enriched in the pathways. The colors of each bubble are determined based on the *P-value* of the identified pathways. Size of the bubble represents number of DEGs enriched in the pathways. The figures were drawn using R and ggplot package.

**Fig 4:**
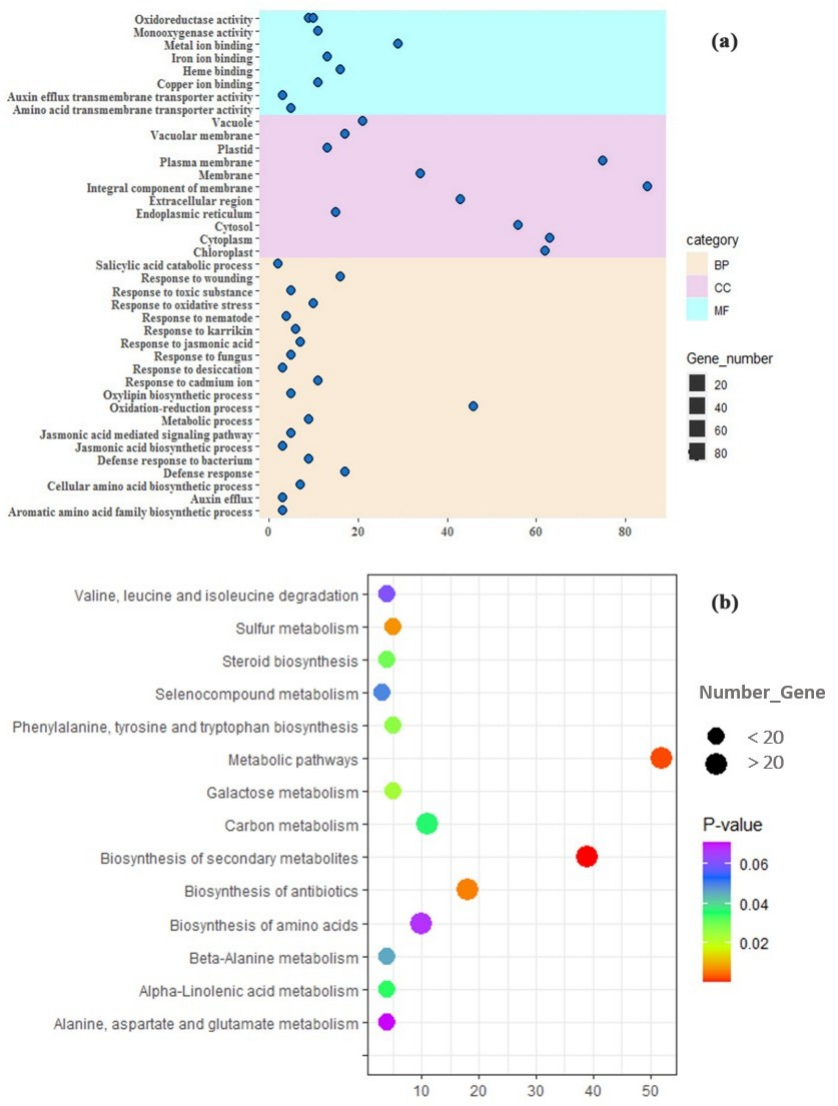
Gene enrichment and pathway analyses of hormonal DEGs. (a) Shows gene ontology analysis using DAVID. The most significantly (*P-value* ≤ 0.05) enriched GO terms in biological process (BP), molecular function (MF) and cellular component (CC) categories related to signaling pathways are presented. The horizontal axis shows the number of genes based on the *P-value*, while the vertical axis represents Go terms. (b) Shows KEGG pathway analysis. Y-axis shows the pathways associated with hormonal signaling and the X-axis shows the number of DEGs enriched in the pathways. The colors of each bubble are determined based on the *P-value* of the identified pathways. Size of the bubble represents number of DEGs enriched in the pathways. The figures were drawn using R and ggplot package.

Finally, 1232 biotic DEGs and 308 hormonal DEGs were studied by GO analysis (Figs 3 and 4). The GO of biotic DEGs (Fig 3A) showed that the top enriched GO terms in BP include the process of oxidation-reduction (8.4% genes), regulation of translation (6.4% genes), response to cadmium ion (3.9% genes), ending of embryo development in seed dormancy (3.5% genes), folding of the protein (2.8% genes), and system of transport (2.8% genes). The most important mechanisms were related to response to ABA (2.6% genes), photosynthesis (2.5% genes), defense against bacterium (2.4% genes), cytokinin (CK) (1.9% genes), ET (0.9% genes), oxidative stress (0.8% genes), JA biosynthetic process (0.7% genes), hydrogen peroxide catabolic process (0.7% genes), and reactive oxygen species (0.4% genes) (Fig 3A). These mechanisms trigger the expression of genes associated with pathogenesis and prevent further infection or actively inhibit pathogen reproduction [89]. In response to pathogens, hormones are the central messengers. Gene-specific hormones including *downy mildew resistance 6* (*DMR6*), *PAL1*, *lectin-like oxidized low-density lipoprotein receptor-1* (*LOX1*), *JAZ1*, *arginine decarboxylase 2* (*ADC2*), *12-oxophytodienoate reductase 2* (*OPR2*), *acyl-CoA-binding protein 4* (*ACBP4*), *PR4*, *mitogen-activated protein kinase 6* (*MPK6*), and *multiprotein bridging factor 1a* (*MBF1a*) are involved in defense mechanisms. In addition, specific responsive genes to ABA (*phytoene dehydrogenase* (*PDS3*), *carotenoid isomerase* (*CRTISO*), and *geranylgeranyl diphosphate synthase 1* (*GGPS1*)), auxin, and CKs (*salivary peroxidase* (*SAPX*), *magnesium-chelatase subunit ChlI-1* (*CHLI1*), and *AT2G44060*) have also been suggested to have key roles in regulating plant defenses. It has been shown that CK enhances resistance against necrotrophic and biotrophic pathogens such as Xanthomonas through a process that relies on SA and ET signaling [90].

The KEGG pathway analysis for biotic DEGs revealed significant enrichment in pathways associated with the biosynthesis of secondary metabolites, antibiotics, and amino acids (Fig 3B).

In addition, the most percentage of hormonal DEGs (Fig 4A) were involved in the oxidation-reduction process (15% genes), defense response (5.5% genes), and the responses to oxidative stress (3.2% genes) in BP. In MF, metal ion binding and oxidoreductase activates were dominant. The category of CC and KEGG pathway analysis was the same as the components of biotic DEGs (Fig 4B).

## Co-expression analysis and module identification

The R package WGCNA was applied to 1232 biotic and 308 hormonal DEGs to identify co-expressed modules. The soft-threshold power was one of the main parameters in WGCNA, making the constructed network more in line with the scale-free network characteristics and average connection degree of the gene co-expression module. When the soft-thresholding β power was 10, the scale-free topology fitting index (*R2*) was optimized to ≥ 0.8, and this power was selected to build a hierarchical clustering tree. By the dynamic tree-cutting algorithm, DEGs were grouped into 6 modules for biotic data ranging from 81 to 431 genes per module (S3A Fig and Table 2) and 7 modules for hormonal data ranging from 141 to 926 genes per module (S3B Fig and Table 3). Finally, each module was assigned on a unique color label, which was used as an identifier for the analyses.

**Table 2:** Modules of biotic stress studies.

Module name	Genes number
Blue	296
Brown	229
Green	81
Red	68
Turquois	431
Yellow	124

**Table 3:** Modules of hormonal treatment studies.

Module name	Number genes
Blue	926
Brown	305
Green	206
Red	142
Turquois	947
Yellow	221
Black	141

**Fig 5:**
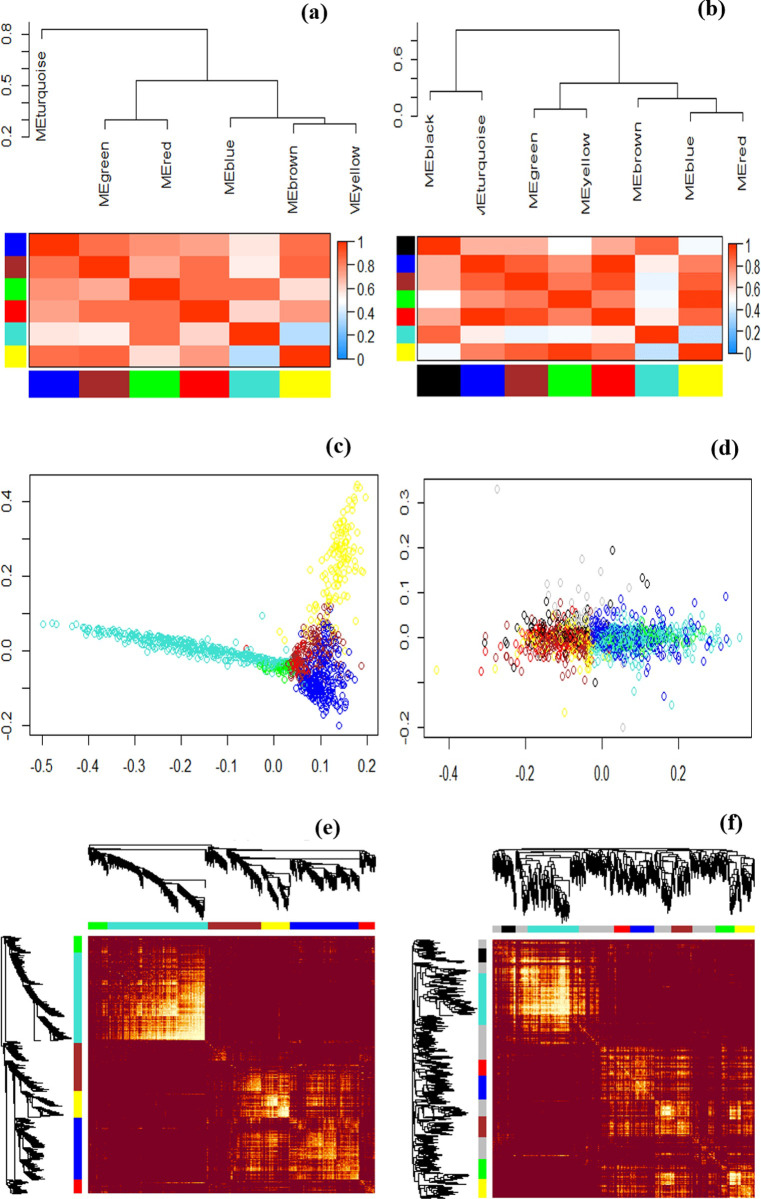
Co-expression analysis and modules identification. a, b) The module eigengene adjacency is showed by hierarchical clustering and heat map. In the heatmap, each row and column correspond to a specific module’s eigengene was denoted by module color. Within the heatmap, red indicates high adjacency (positive correlation) and blue shows low adjacency (negative correlation) as shown by the color legend. A module eigengene summarizes the gene expression profile of each module for (a) biotic and (b) hormonal studies. In the heatmap of module-module relationships, the progressively more saturated blue and red colors indicate the high co-expression interconnectedness. c-d) Multi-dimensional Scaling (MDS) map of each gene located in a module of the c) biotic or (d) hormonal studies. MDS plot demonstrating the similarity of gene expression patterns between different modules. Genes of different modules are marked in different colors. e-f) show network heatmap plots. Co-expression network modules of (e) biotic or (f) hormonal DEGs are presented. In the TOM plot, darker red color represents low overlap as progressively light color represents higher overlap among DEGs. Blocks of darker colors along the diagonal correspond to the modules. Gene dendrograms and module assignments are also displayed on the left side and the top.

The hierarchical clustering was illustrated in the following. As the dendrogram demonstrated, we achieved two clusters, including 4 sub-clusters for biotic data (Fig 5A) and 5 sub-clusters for hormonal data (Fig 5B). In most modules, including blue, brown, and yellow or red and green modules, biotic DEGs were expressed similarly according to Multidimensional Scaling (MDS) (Fig 5C). In addition, hormonal DEGs in the modules of blue, green, and turquoise or brown, red, and yellow exhibited a similar expression pattern (Fig 5D). The brown and blue modules showed the strongest gene-gene interconnected based on TOM dissimilarity distances.

In the eigengene adjacency heatmap, the slope of the variance in color from black or blue to yellow represents the connectedness of genes for different modules from strong to weak, whilst the red highlights are the significant modules related to biotic and hormonal data (Fig 5A and 5B). The brown and blue modules exhibit the strongest gene-gene interconnected based on TOM dissimilarity distances. The adjacency heatmap of eigengenes module represents the summary of the results in the form of color-coded tables, illustrating the good agreement between consensus modules of each dataset or between specific and common modules of biotic and hormonal datasets. It reveals that most modules, especially the brown and blue modules, are preserved in biotic and hormonal treatment. Moreover, based on the TOM, light-yellow color represents a higher overlap between modules, and a progressively dark color represents a lower overlap between modules. Blocks of light colors along the diagonal are the modules (Fig 5E and 5F).

Finally, the gene enrichment analysis was performed for BP category to understanding the biological functions associated with the modules (S3 Table). Based on the BP results of biotic modules, the turquoise module was significantly enriched in oxidation-reduction processes, and the blue module was enriched in intracellular protein transport and photosynthesis. The brown, yellow, green, and red modules were related to cadmium ion response, translation, protein ubiquitination, and stress response, respectively.

According to the GO results of hormonal modules (S3 Table), the most important BP terms enriched in turquoise and blue modules were significantly enriched in photosynthesis, response to cytokinin, protein transport, response to cadmium ion, protein folding, isopentenyl diphosphate biosynthetic process, oxidation-reduction process, and ribosome biogenesis. The brown module was related to the defense response to a bacterium, reactive oxygen species metabolic process, oxidation-reduction process, response to wounding, and metabolic process. In addition, 600 common genes (17.1%) were identified between biotic and hormonal modules in which the turquoise (195 genes), blue (169 genes), and brown (117 genes) modules involved the most common genes.

JA and SA regulate the DEGs in these modules in the same or opposite direction. For instance, the JA/SA co-induced the genes *amine oxidase copper containing 3* (*AOC3*), *JAZ1*, *overexpressor of cationic peroxidase 3* (*OCP3*), *redox regulators glutathione* (*GRX480*), and *RP1* in turquoise and blue modules, while JA suppresses the SA-induced immune responses by inhibiting the expression of *PRs*.

### Hub genes

**Fig 6:**
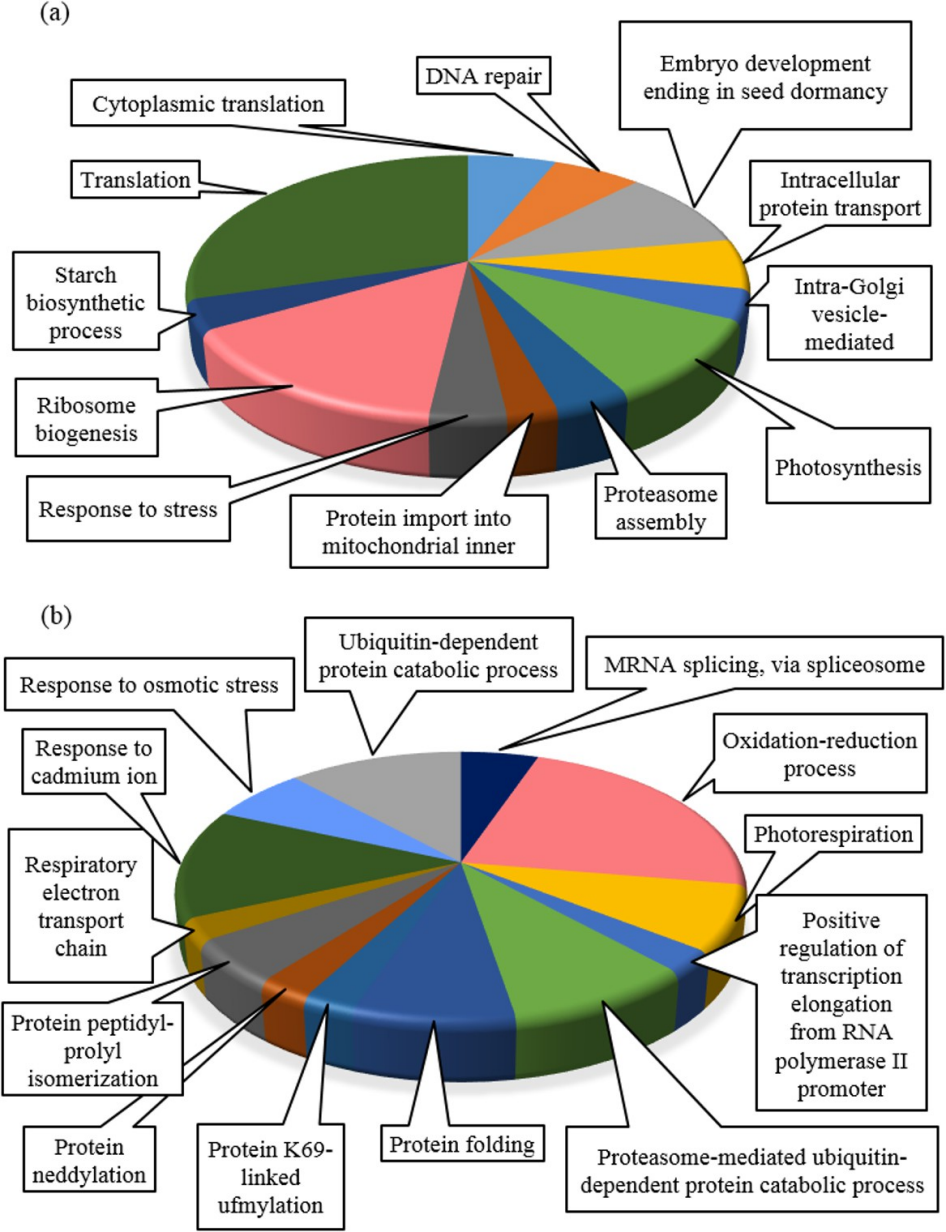
GO analysis of hub genes (*P-value* ≤ 0.05). (a) Biotic hub genes. (b) Hormonal hub genes.

The top 30 hub genes were chosen for each module. GO analysis was performed for 177 biotic and 198 hormonal hub genes. The hub genes related to biotic modules were strongly enriched in photosynthesis, translation, and ribosome biogenesis pathways (Fig 6A).

Among the hormonal modules, the top enriched BP terms were associated with the oxidation-reduction process, response to cadmium ion, and ubiquitin-dependent protein catabolic process (Fig 6B). In the following, 10 common hub genes (2.7%), such as *NF-YC2*, SSI2, *ARAC1*, and *PFD1*, were identified between biotic and hormonal data (S4 Fig). Besides, hub genes with unknown functions, including *AT3G26670*, *AT1G67620*, *AT1G05720*, *AT1G52600*, *AT5G59140*, and *AT3G22290*, were identified as potential candidates for further investigation.

### Identification of transcription factors

**Fig 7:**
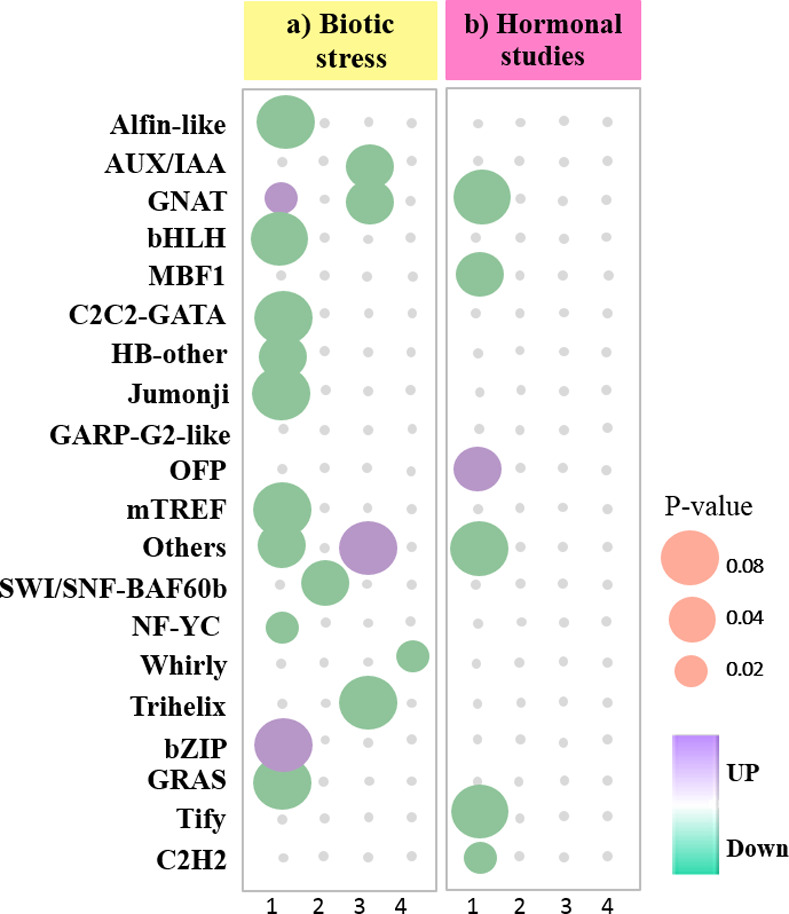
TF families identified in DEGs. (a) The number of up- and down-regulated biotic TF families. (b) The number of up- and down-regulated hormonal TF families. The Y-axis refers to the number of genes. The X-axis shows TF families.

To identify important TFs, DEG sequences were used to the BLASTX against the iTAK database. All 24 TFs found for biotic DEGs belonged to 16 different TF families and were either directly or indirectly involved in hormonal signaling and response to biotic and abiotic stresses (Fig 7A and S5 Table). Members of the auxin/indole-3-acetic acid (Aux/IAA), GCN5-related *N-*acetyltransferases family (GNAT), nuclear factor Y, subunit C (NF-Y), and WHIRLY families were the top classes. Among these TFs, only 5 families, including bZIP and GNAT, were significantly up-regulated, while most family members were down-regulated. These TFs were more involved in response to wounding, bacterium, karrikin, oxidation-reduction, ABA, JA, and in the arginine biosynthetic process. We found a higher abundance of TFs in the turquoise module (8 TFs) as compared to the brown (7 TFs), green (4 TFs), blue (2 TFs), and red (2 TFs) modules. The WHIRLY (in the turquoise module), GNAT, and NF-YC families (in the green module) were also considered as the hub genes (S5 Table).

Furthermore, 6 TFs related to hormonal DEGs were identified, belonging to 6 different TF families (Fig 7B and S5 Table). Among hormonal TF families, only one family of OVATE family proteins (OFPs) was significantly up-regulated, while other families were down-regulated. These TF families were more involved in response to oxidative stress, wounding, bacterium, ABA, and JA signaling pathways. We further extended the study to detect hub genes and TFs in each module. The brown module was enriched for Tify and OFP TF families, whereas the turquoise, red, and black modules included at least one member of GNAT and MBF1 families.

Among the identified TF families of biotic or hormonal studies, there was only one common significantly up-regulated TF of *TIFY10A* (*AT1G19180*) that was involved in response to JA, defense responses to the bacterium, and regulation of defense responses.

### Identification of PKs

PKs are important signaling regulators in response to environmental stresses [91]. To identify PKs, DEG sequences were used to the BLASTX against the iTAK database. Three up-regulated PKs were identified among the biotic DEGs and classified into RLK (2 types of receptor-like kinase-Pelle (RLK-Pelle), and tyrosine kinase-like kinase (TKL) (families (Table 4). The RLK-Pelles, the largest group, were commonly identified in turquoise and blue modules. In addition, TKL-Pl-4 was identified in the turquoise module.

**Table 4:** PKs identified in biotic and hormonal treatment studies.

Family	Count	Accession	Up/Down	Module	Dataset
TKL-Pl-4	1	Contig11835_at (*AT5G58950*)	Up	Turquoise	Biotic
	1	Contig11835_at (*AT5G58950*)	Up	Red	Hormonal
	1	Contig18153_at (*AT4G35780*)	Down	Turquoise	Hormonal
RLK-Pelle_LRR-XI-1	1	Contig15860_at (*AT1G34420*)	Up	Blue	Biotic
RLK-Pelle_SD-2b	1	Contig18914_at (*AT4G32300*)	Up	Turquoise	Biotic
RLK-Pelle_SD-2b	1	Contig22773_at (*AT5G60900*)	Up	Black	Hormonal

Among hormonal DEGs, three PKs were identified, which were classified into RLK-Pelle and TKL families (Table 4). Among the identified TKLs, there was one up-regulated *(AT5G58950*) gene at the red module and the others were down-regulated. RLK-Pelle families were also identified in the black module. There was only one common up-regulated PK detected in the red and turquoise modules among biotic and hormonal PKs. This PK is TKL-Pl-4 (*AT5G58950*).

### Identification of miRNAs

**Fig 8:**
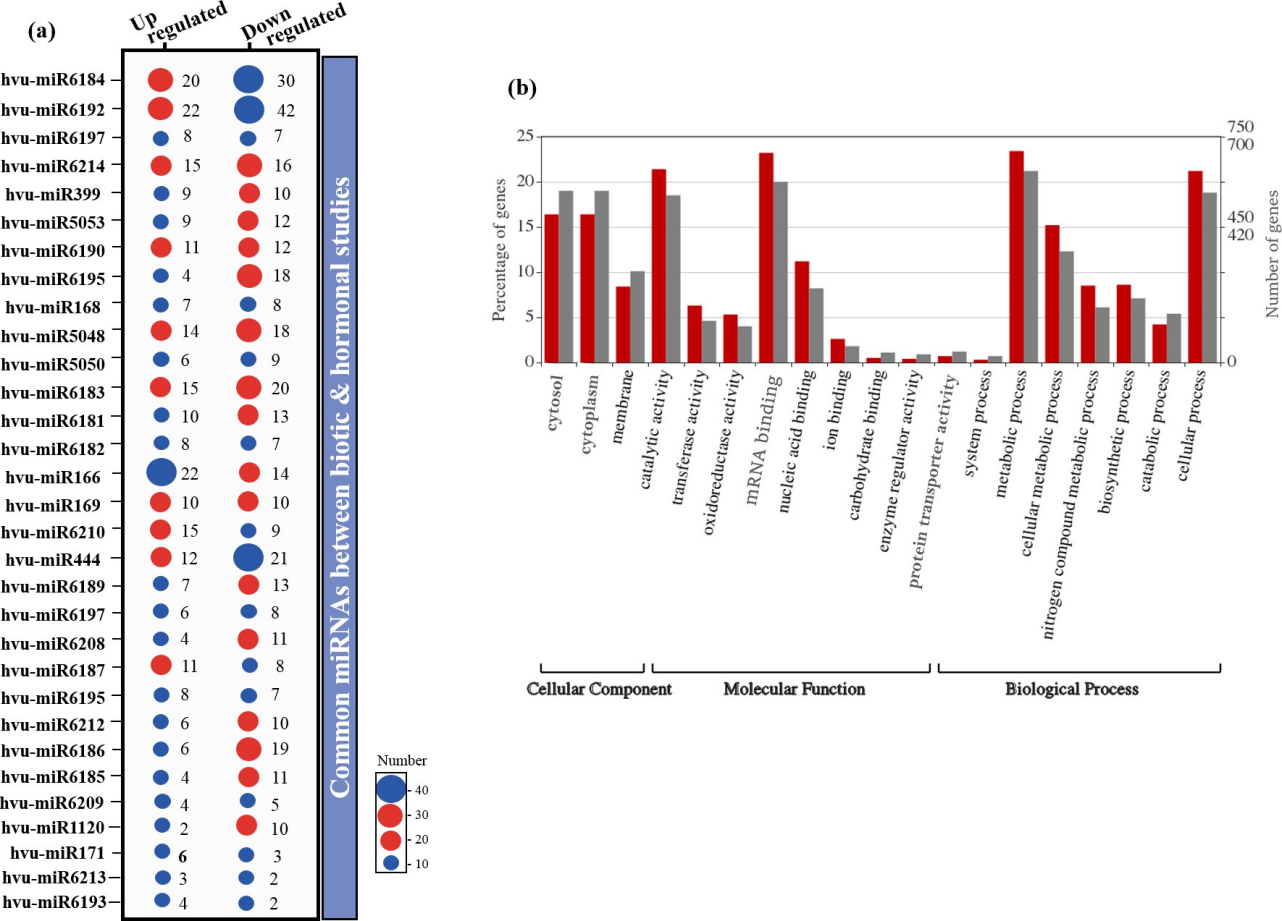
Identification of miRNAs and their targets. (a) Common miRNAs between biotic and hormonal studies are showed. The Y-axis refers to the number of miRNAs. The X-axis shows miRNA families. (b) Shows GO classification for the target genes of differentially expressed miRNAs. Categorization of miRNA target genes with WEGO for biotic and hormonal studies are presented. The horizontal axis is the GO classification; the vertical is the percentage of genes (left) and number of genes (right). The miRNAs are divided into GO categories. The genes targeted by the entire miRNAs: red column, the genes targeted by the differentially expressed miRNAs: gray column.

The psRNATarget tool was used for predicting potential miRNAs that target the DEGs. Among the biotic DEGs, 432 miRNAs belonging to 56 conserved families were found (S6 Table). Furthermore, 85 miRNAs belonging to 32 conserved families were identified among hormonal DEGs (S6 Table). It was found that 15 target genes were common across both datasets; of which miR6192 was the most frequent (Fig 8A).

All the putative target genes such as *Ribosomal Protein S1* (*RPS17*), *ROF2* (*FKBP65*), *Eukaryotic Translation Initiation Factor 3 Subunit E* (*EIF3E*), *RNA polymerase I-associated factor* (*PAF67*), *Abnormal Inflorescence Meristem* (*AIM1*), and *PKT3* were GO categorized. The results showed that these genes were involved in different MFs, i.e., oxidoreductase, transferase, and enzyme regulatory activities, and played roles in many BPs, i.e., metabolic, cellular, and biosynthetic processes (Fig 8B).

### Cis-acting elements

The 1000 bp upstream flanking region of DEGs was used to predict conserved cis-regulatory elements (CREs). For each biotic and hormonal DEG, MEME analysis identified 11 significant cis-acting elements (Tables 5 and 6).

**Table 5:** The conserved cis-acting elements found in the promoter of biotic DEGs by the MEME analysis.

Motif name	*E-value*	Width	Best match in JASPAR and PLACE	Significant GO term identified by GOMO	Signaling pathway
Motif 1	7.2e-083	29	MA1267.1	CC: chloroplast	ABA, GA, IAA, JA, and SA
Motif 2	8.3e-035	15	MA1267.1	-	ABA, GA, IAA, JA, and SA
Motif 3	8.6e-031	21	MA1267.1	CC: plasma membrane MF: TF activity	ABA, GA, IAA, JA, and SA
Motif 4	1.5e-013	29	MA1267.1	MF: TF activity BP: regulation of transcription CC: endomembrane system, plasma membrane	ABA, GA, IAA, JA, and SA
Motif 5	2.5e-006	48	MA0657.1	MF: TF activity CC: nucleus, plasma membrane MF: protein binding BP: regulation of transcription	JA and MeJA
Motif 6	1.0e-004	41	MA1267.1	MF: TF activity, protein binding, serine/threonine, kinase activity CC: nucleus, plasma membrane	ABA, GA, IAA, JA, and SA
Motif 7	2.9e-002	29	MA1415.1	MF: TF activity CC: nucleus, plasma membrane BP: regulation of transcription, MF: protein binding	JA and SA
Motif 8	2.1e-001	11	MA1268.1	-	ABA, GA, IAA, JA, and SA
Motif 9	3.8e-002	39	MA1327.1	-	JA
Motif 10	1.4e+000	29	MA1354.1	MF: TF activity CC: plasma membrane	JA
Motif 11	1.3e+001	15	MA1267.1	CC: plasma membrane, nucleus BP: transmembrane receptor protein tyrosine kinase signaling pathway MF: TF activity, protein serine/threonine, kinase activity	ABA, GA, IAA, JA, and SA

**Table 6:** The conserved cis-acting elements found in promoter of hormonal DEGs by the MEME analysis.

Motif name	*E-value*	Width (bp)	Best match in JASPAR and PLACE	Significant GO term identified by GOMO	Signaling pathway
Motif 1	5.7e101	21	MA1267.1	MF: TF activity CC: nucleus, plasma membrane BP: regulation of transcription	ABA, GA, IAA, JA, and SA
Motif 2	1.6e-074	50	MA0150.2	MF: TF activity CC: nucleus, plasma membrane	SA
Motif 3	8.2e-071	50	MA0072.1	MF: TF activity, protein serine/threonine kinase activity CC: nucleus, plasma membrane BP: regulation of transcription, DNA-dependent	JA and MeJA
Motif 4	3.0e-069	50	MA1268.1	CC: chloroplast BP: regulation of transcription MF: TF activity	ABA, GA, IAA, JA, and SA
Motif 5	2.1e-068	50	MA1277.1	CC: chloroplast	ABA, GA, IAA, JA, and SA
Motif 6	6.4e-058	50	MA1260.1	-	ET, ABA, CK, and JA
Motif 7	9.0e-058	21	MA1267.1	-	ABA, GA, IAA, JA, and SA
Motif 8	3.1e-054	50	MA0777.1	-	JA
Motif 9	4.5e-054	50	MA0054.1	MF: TF activity	JA
Motif 10	1.2e-053	50	MA0578.1	-	JA and MeJA
Motif 11	9.0e-053	50	MA0415.1	-	SA

Among the top motifs for biotic and hormonal DEGs, motifs 1 and 4 showed the highest frequency. Moreover, most of the cis-acting elements have related to C2H2 zinc finger motifs like A1267.1, MA1267.1, MA1267.1, and MA1268.1 for the biotic DEGs, as well as MA1267.1 and MA0415.1 for the hormonal DEGs (S7 Table). The GOMO analysis for the motifs found by MEME detected many interesting BFs (Tables 5, 6 and S8). GO term analysis of biotic DEGs indicated that these motifs were involved in the regulation of transcription, DNA-dependent, and signaling pathways. WRKY families targeted the main biotic motifs, whereas 85% of the DEGs involved at least one WRKY binding site. Moreover, these motifs were involved in MFs of TF and protein serine/threonine kinase activities (Table 5). GO term analysis of hormonal DEGs showed that the motifs were mainly involved in the regulation of transcription (Table 6). The key TF families comprised WRKYs, MYBs, bHLHs, and bZIPs. Finally, this analysis identified the motifs related to SA (GO: 0009751) and auxin pathways (GO: 0009733) (S9 Table).

### Protein-protein interactions and selection of key genes

**Fig 9:**
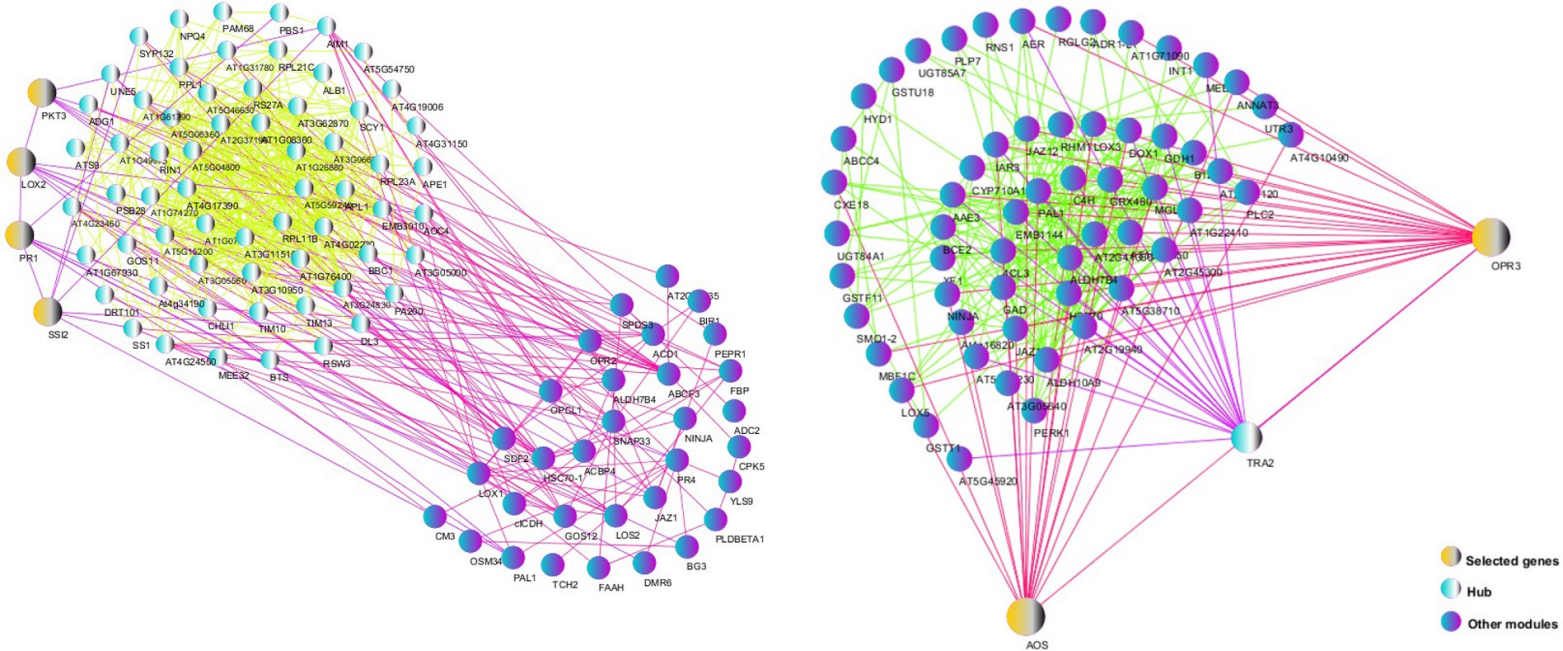
The protein-protein interaction network of hub genes and DEGs using STRING database and Cytoscape software. (a) The network of interactions among biotic hub genes and the DEGs is showed. (b) The network of interactions among hormonal hub genes and the DEGs is presented. The yellow nodes represent the selected key genes for qPCR validation, blue nodes represent the hub genes, and the purple nodes represent the most connected genes to the key genes in the network.

It is important to find genes that are regulated for any type of biotic stress or hormonal treatment. We selected 1232 biotic DEGs and 308 hormonal DEGs. The DEGs also represent common responses to defense and may be useful in describing general responses to the biotic stresses in barley and Arabidopsis. For this purpose, the analysis of protein-protein interactions (PPIs) using the STRING database and Cytoscape software was performed (Fig 9A and 9B).

The network was plotted for 265 key biotic DEGs, which were involved in the most important mechanisms responsive to the biotic stresses, and 67 hormonal key DEGs, which were related to the hormonal signaling pathways (Table 7).

**Table 7:** The most important molecular mechanisms related to the biotic and hormonal studies.

Selective mechanisms related to the hormonal data		Selective mechanisms related to the biotic data	
Number of genes	Meaningful mechanisms	Number of genes	Meaningful mechanisms
15	JA biosynthetic process	104	Oxidation-reduction process
5	Response to fungus	9	Hydrogen peroxide catabolic process
17	Defense response	12	Response to ET
3	Auxin	6	Response to reactive oxygen species
6	Response to karrikin	11	Oxidative stress
9	Defense response to bacterium	43	Response to pathogens
2	SA catabolic process	24	Response to CK
4	Response to nematode	40	Photosynthesis
6	Response to ET	16	JA biosynthetic process

A list of genes that showed the most protein-protein interactions was prepared (S10 Table). It is interesting to notice that some up-regulated biotic or hormonal hub genes may play a key role in response to fungal pathogens or hormonal signaling. Finally, 4 biotic key genes, including *PKT3*, which was active in the local and systemic induction of JA biosynthesis after pathogen attack, *PR1*, which involved in response to a variety of pathogens and a useful molecular marker for the SAR signaling, *SSI2*, which was active in SA- and JA-mediated defense signaling, and *LOX2*, which was active in the pathogen resistance and synthesis of JA, were identified. In addition, there were two hormonal key genes, including *OPR3* and *AOS*, which involved in the biosynthesis of JA and other oxylipin signaling molecules, as well as the response to fungal pathogens.

**Fig 10:**
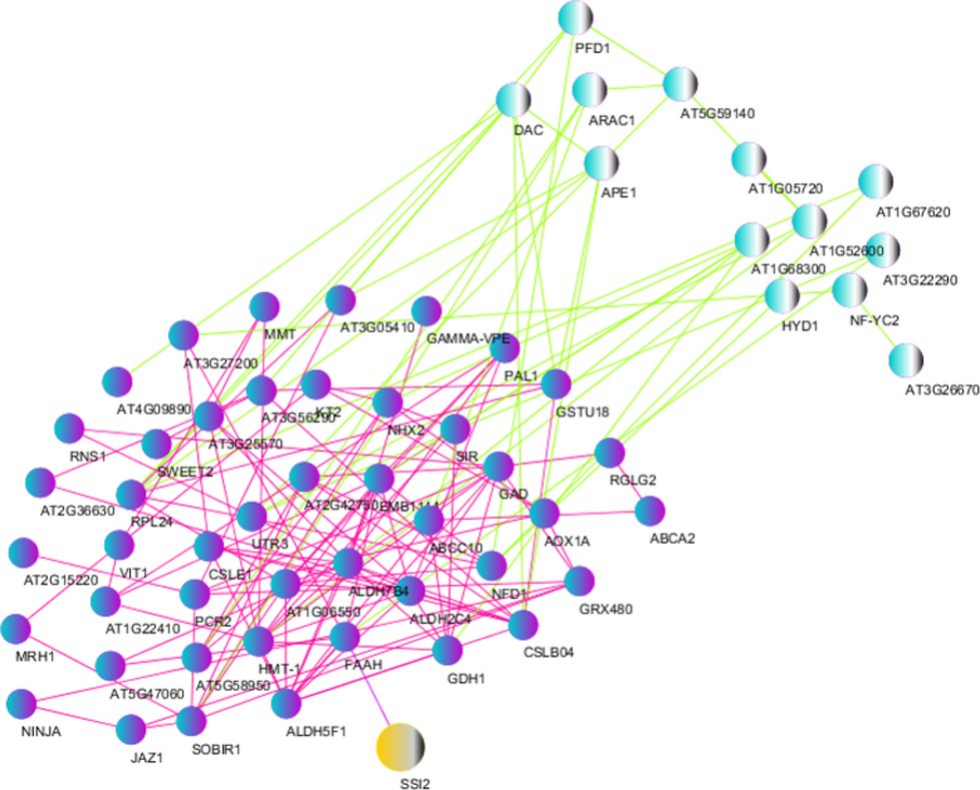
The protein-protein interaction network of common DEGs using STRING database and Cytoscape software. Interaction network of 61 common DEGs between hormonal and biotic datasets. The yellow colored nodes represent the key genes expressed in the pathways of resistance to the pathogens and signaling of hormones, and the pink colored nodes represent the genes with the most connectivity to major genes in the network.

Finally, a PPI network was illustrated to study key genes that are commonly expressed among biotic and hormonal DEGs. We showed a total of 61 common DEGs such as *SSI2*, *PAL*, *JAZ1*, *APE1*, *ribosomal protein L24* (*RPL24*), and some others significant DEGs especially with unknown function which are simultaneously active in the pathways of resistance to the pathogens and signaling of hormones (Fig 10).

#### Validation of candidate genes

**Fig 11:**
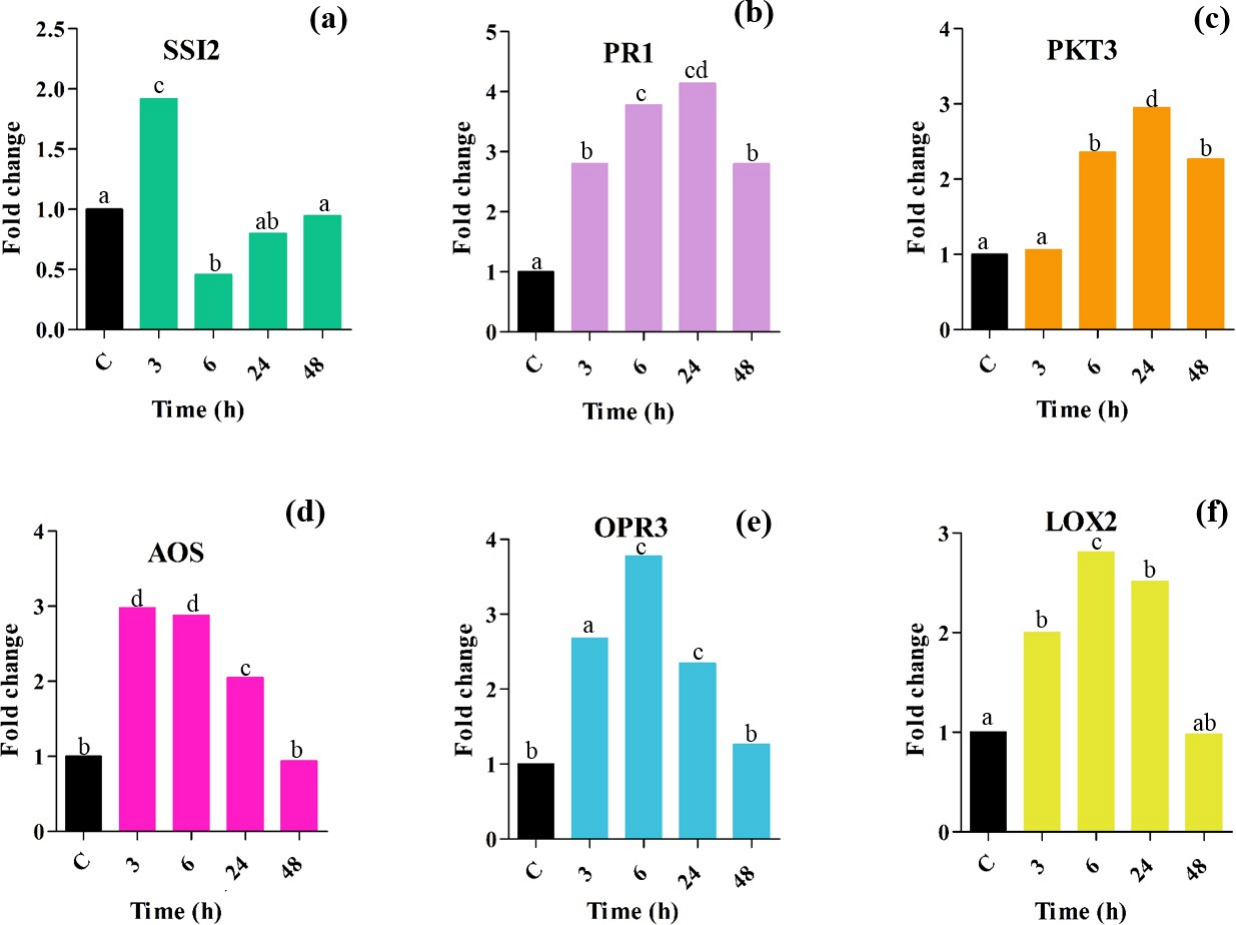
Relative expression analysis of hub genes. The expression of hub genes at the time points of 3 h, 6 h, 24 h, and 48 h after 100 μM MeJA treatment. The different letters on the columns represent the significant differences given by Duncan’s multiple range statistical analysis. Vertical bars indicate ± SE of the mean (n = 3, *P-value* ≤ 0.05). The X-axis shows the times after MeJA treatment.

Here, a significant number of all DEGs were regulated by JA/SA in the same or opposite direction. In this study, 238 co-induced JA/SA DEGs were defined as wide-spectrum pathogen resistance DEGs activated by bacteria, fungi, viruses, and aphids (S10 Table and Figs 9 and 10). To experimentally validate the reliability of the DEGs obtained from the integrative transcriptome analyses, a total of 6 hub genes including *SSI2*, *PR1*, *PKT3*, *AOS*, *OPR3*, and *LOX2* were candidates for qPCR (Figs 11 and S6). These genes were chosen based on being highly differentiated in response to the MeJA and pathogens.

The candidate genes (*P-value* ≤ 0.05) responded positively or negatively to the application of MeJA. However, the expression level of *SSI2* reached the highest peak at 3 h, about 2 times higher than the control. Notably, *SSI2* showed a dramatic suppression within 6 h after treatment, and it showed no significant increase after 24 to 48 h compared to the control, indicating that the *SSI2* may perform different functions in response to MeJA signaling (Fig 11A).

Conversely, we found that the expression of *PR1* and *PKT3* were significantly increased (Fig 11B and 11C). Although the FC at the time point of 48 h slightly decreased compared to the rest of the periods, it showed a significant increase in the expression compared to the control. Meanwhile, for the *AOS*, *OPR3* and *LOX2* expression, we found that they were significantly induced in response to MeJA treatment after 24 h (Fig 11D–11F). Additionally, the expression of *AOS*, *OPR3* and *LOX2* was induced at 3 to 24 h after treatment, while it decreased to its basal level (restored to normal condition) after 48 h (Fig 11D–11F).

## Discussion

In spite of many researches, there has not been sufficient clearance of the molecular mechanisms of defense in barley. For instance, what are the main mechanisms of hormonal signaling against different pathogens or what are the main gene networks? The integrative analysis of the transcriptome is one of the most efficient approaches to finding the answer to biological questions, thus enabling researchers to achieve the huge amounts of valuable information. In this study, based on the biotic stress and hormonal treatment data, we discussed the defense responses of barley and several specialized pathways related to pathogens. In addition, the responsive specific novel genes to defense hormones in barley that are the results of our research were elucidated.

### Common and unique biological processes responsible for pathogen resistance and JA signaling

To identify the main DEGs and key pathways, the integration of transcriptomic data retrieved from various biotic stress and hormonal treatment studies in barley, was applied by meta-analysis. Pathogen resistance and hormone signaling are studied using this approach, which allows us to understand the response pathways between pathogens and hormones. The meta-analysis identified 1232 biotic and 308 hormonal DEGs (Fig 2A and 2B and S2 Table). Of 61 common DEGs between biotic and hormonal studies, 1.2% and 0.8% were categorized as up- and down-regulated, respectively (Fig 2B).

The gene enrichment analysis suggested that DEGs were enriched in various BPs (Figs 3 and 4). The terms of photosynthesis, response to cadmium ion, protein folding, oxidation-reduction process, response to CK, defense response to the bacterium, defense response to fungus, cellular response to oxidative stress, response to ABA, JA mediated signaling pathway, response to reactive oxygen species, response to wounding, and response to biotic stresses and hormones, were also found to be significant. For example, PAL is involved in the phenylpropanoid pathway as the primary biosynthetic enzyme and in SA biosynthesis during SAR induction [92, 93]. The importance of SA as a key signaling hormone in resistance to diseases has considerable interest. Since SA accumulation is associated with the expression of defense marker genes, the *PAL* pathway produces the intermediates required for the biosynthesis of not only SA but also many other secondary metabolites, such as lignin [94]. *PAL* transgenic crops were more resistant to brown planthopper (BPH) [92]. Hence, the expression of some *PALs* was significantly up-regulated under pathogen infestation, the up-regulated *PALs* may be correlated with BPH-elicited phenolamide and flavonoid accumulation in crops. Carbohydrate metabolism has been widely shown as an essential pathway influenced by biotic stress responses [95]. Growing organs and tissues are the complex sinks of carbohydrates attracting photosynthesis energy produced by leaves. The source and sink relationships allow the allocation of carbon during a variety of stresses to improve plant performance [96]. The disruption of source-sink connections has been associated with the early pathogenic mechanisms of diseases in plants [97]. The dysregulation of this pathway at the transcriptomic level may be associated with a general plant stress state.

Plant elicitors are attractive and promising factors for plant resistance because they increase host endogenous immunity without direct toxicity to the pathogens [98]. Screening strategies for these elicitors could be mentioned as pathogen-responsive-gene-based (PRGB) approaches. Here, 4% of hormonal (most JA/SA) induced DEGs are commonly regulated by biotrophic and necrotrophic pathogens, which were confirmed by microarray datasets, and were defined as broad-spectrum disease resistance genes (Fig 2). These DEGs are involved in nitrogen storage, photosynthesis, auxin transport, and gibberellin biosynthesis. It suggested that plants under treatment of the elicitors or attack by pathogens have prioritized appropriate defense response over the growth by activating the shared SA and JA responses. The results showed that the JA-responsive marker gene *PDF1*.*2* and SA-responsive marker gene *PR1* were uniquely upregulated by JA and SA, respectively. The promoter of *PDF1*.*2* responded to MeJA but not to SA [99]. Thus, certain downstream genes of SA signaling might be free from the impact of JA signaling activation. Therefore, plants simultaneously activate JA/SA signaling pathways to suitable responses to the different pathogens [100]. The *allene oxide cyclase* (*AOC4*), *JA carboxyl methyltransferase* (*JMT*), *AIM1*, and *PKT3* families are the regulators of seed germination and subsequent seedling growth. Moreover, they have required for the local and systemic induction of JA and MeJA biosynthesis after wounding. It seems to be involved in JA biosynthesis during senescence [101–104].

The study of the hormonal responses to pathogens is vital because the hormonal signaling pathways regulate defense responses antagonistically or synergistically [105]. It is observed that several genes (such as *WRKY* families) involved in auxin, ET, JA, and SA signaling were usually affected against all types of pathogens. Fungal pathogens up-regulate ET, benzyl-adenine (BA), and SA signaling pathways while mostly repressing JA-related genes. For example, *Erwinia amylovora* up-regulates ET and GA signaling genes, while viruses affect some essential genes involved in ET and auxin signaling [105]. Our results confirmed that all barley pathogens deeply affected the hormone-related pathways.

### TFs, regulatory factors in response to pathogens

To present the information regarding how genes are regulated in barley against pathogen attacks and hormone treatments, we identified more important TFs. TFs only not act as the activators for the expression of stress-inducible genes but also play a significant role in signal transduction pathways in biological networks and promote plant adaptation to various environmental conditions [106]. Finally, we identified 24 biotic TFs belonging to 25 TF families and 6 hormonal TFs belonging to 6 TF families (S5 Table). The major members of TF families, such as GNAT, NF-YC, and WHIRLY, were the top classes and were also considered hub genes (Fig 7). In this analysis, *WHIRLY* was up-regulated. Plants have a small family of single-stranded DNA-binding proteins called WHIRLY, which are also involved in defense gene regulation [107]. In barley, WHIRLY binds to the promoter of the *HvS40* senescence-related gene, which is induced by pathogens, senescence, and hormones, such as SA, JA, and ABA [107, 108]. Transgenic barley with suppressed *WHIRLY1* remained almost green phenotype despite having higher SA than the wild-type [109]. The plant-specific *WHIRLY* is induced by SA and required for both SA-dependent disease resistance and SA-induced SAR gene expression. WHIRLY is required for both full basal and specific pathogens resistance responses. It has been found that *NPR1* is required for SAR, and that is an important component of the SA signaling pathway that is activated independently of the key SAR regulator *NPR1*. However, both *NPR1* and *WHIRLY1* are required for optimal SA-induced defenses. WHIRLY1 contributes significantly to pathogen resistance in SA-dependent lines [110].

The only one identified *NF-Y* was down-regulated (Fig 7). It has shown that an increasing number of *NF-Ys* play significant roles in the various stages of root node coexistence and arbuscular mycorrhizae as well as in the interaction of plants with pathogenic microorganisms [111]. Barley *NF-Ys* comprise a relatively diverse gene family involved in various processes such as starch metabolism, sucrose metabolism, and photosynthesis. There is less evidence about the role of *NF-Ys* against pathogenic attacks. Overexpression of rice *NF-YA* (*OsHAP2E*) causes resistance to *Magnaporthe oryzae* and *Xanthomonas oryzae* [112]. The signaling pathways of the *NF-YAs* regulate beneficially host-microorganism interactions and indicate the existence of common mechanisms between both types of biological interactions. The results demonstrate that some members in the miR169 family might handle NF-Ys to activate the ISR pathway [113]. Using these findings, we can illustrate the contrast between regulators allowing plants to construct complex networks of interacting pathways in the presence of various pathogens and hormonal signals.

The GNAT family is generally considered to be comprised of four subfamilies designated GCN5, ELP3, HAT1, and HPA2. It has shown that members of the GNAT superfamily activate gene transcription by the promotion of euchromatin formation and exert various levels of contributions to cell morphogenesis and virulence [114]. In addition, a GNAT acyltransferase has been identified that plays a role in multiple biological processes, including the regulation of growth, morphogenesis, cellulase expression, and pathways related to biotic stress responses and hormone signaling. For example, in *Beauveria bassiana*, three HATs, including a GNAT superfamily member, have previously been characterized to variously affect developmental and virulence pathways in this fungus.

In total, analyses revealed that most TF families such as AP2/ERF, bHLH, bZIP, WHIRLY1, and NF-Ys are not only implicated in the regulation network of SAR or ISR but also are involved in the regulation of SA and ET/JA cross-talks [115]. Transcriptional regulator *NPR1* has been reported to be required for basal resistance toward biotrophic and hemibiotrophic pathogens, as well as the development of SAR and ISR [37]. SA-dependent SAR requires a nuclear function of NPR1, while SA-independent ISR requires cytosolic [116, 117] NPR1 localization and involves the JA and ET [118]. Therefore, post-translational modifications of NPR1 appear to play a significant role in modifying transcriptional defense outputs. In particular, we found that WHIRLY1 and NF-Ys in interaction with the NPR1 regulator play a more important role in connection with SAR/ISR pathways, respectively, and hormone-induced defenses thus can be the center of attention and more study.

### PKs in regulation of signaling networks and immune response

PKs are the largest family of molecular switches, which can regulate protein activities and basic cellular functions. However, only a fraction of PKs is functionally characterized even in model plant species. To investigate signal transduction mechanisms in this study, we identified potential PKs that play key roles in signaling networks and plant responses to various biotic stresses and hormonal signaling. We detected 3 PKs among the biotic DEGs and 3 PKs among the hormonal DEGs that are classified into RLK/Pelles and TKL groups (Table 4). RLK/Pelles play important roles from growth regulation to defense response [119]. The dramatic expansion of this family has been considered very important for plant-specific adaptations [119]. We realized that hundreds of *RLK/Pelles* are up-regulated by biotic stresses. In addition, the rate of stress responsiveness is related to the degree of consecutive duplication in RLK/Pelle subfamilies [120]. Members of this family are involved in many different plant processes, including regulation of meristem proliferation, reproduction, hormonal signal transduction, and response to biotic and abiotic stresses [121, 122]. Accordingly, these results suggest that PKs play a central role in signal transduction during plant growth and development and also in plant responses to biotic stresses. One-third of the barley RLK/Pelle members were down-regulated (Table 4) during development, suggesting that these genes may have a negative effect on regulatory functions. Many non-RLK groups, including all of TKLs, however, exhibited high expression levels during development, confirming that these genes may play positive regulatory roles in plant growth. This result shows a positive or negative regulatory function among PKs.

Therefore, RLK/Pelles and TKLs evolved as the families of highly dynamic molecular switches and components of signaling networks, which leads to constitutive activation of the defense response, accumulation of SA, and increase of NPR1 performance [123]. The accumulation of SA can further induce SAR by up-regulating *PRs*. In particular, the fact that the most barley *RLK* families were up-regulated by SA, which are involved in SAR-related gene regulation, makes them interesting candidates for bioengineering broad-spectrum resistance genotypes.

### miRNAs in regulation of gene expression

Plants have evolved complex mechanisms involving a diversity of small RNA-guided stress regulatory networks that can provide new insights for genetically improved plant pathogen resistance. MicroRNAs (miRNAs) are a major class of small non-coding RNAs that have emerged as significant regulators of gene expression during or after transcription [124]. Some miRNAs have been related to pathogen responses, and they play important roles in plants infected by bacteria, viruses, nematodes, and fungi [125].

Barley is one of the most important crops that has been considered for miRNA studies [124]. In the present study, 432 biotic miRNAs belonging to 56 families and 85 hormonal miRNAs belonging to 32 families, and also 15 common miRNAs for both biotic and hormonal data were identified (S6 Table). A high proportion of these miRNAs are involved in the different BPs (Fig 8B). The major members of the miRNAs for both studies belong to conserved families, including *miR6192*, *miR6180*, and *miR6214*, *miR6196*, *miR6184*, *miR166*, and *miR156*. Based on a research by Djami-Tchatchou et al. (2017) about 100 miRNAs were identified for the first time in barley using deep sequencing [124]. As compared these miRNAs to our results, we confirmed that many microRNAs (*miR156*, *miR159*, etc.) showed orthologs in wheat or rice, while it appears that several microRNAs (*miR444*) were expressed in barley specifically.

A combination of small RNA and mRNA degradome analyses to recognition and characterization of many conserved miRNAs (*miR156*, *miR166*, etc.) and novel miRNAs (*hvu-miR1120b*) together with several miRNA target genes were done (Fig 8B). The results showed that some miRNAs are involved in different functions, including carbohydrate translocation, cell differentiation, defense response, photosynthesis, and phytohormone signaling pathways [126]. This finding revealed that miRNAs play a significant role in regulating early growth, the development of barley, and the signaling pathways of different defense hormones [127].

Since ISR differs from SAR in stimulating inducers and signal transduction, different miRNAs may participate in ISR and SAR. *miR393* was the first identified miRNA responsible for triggering the SAR and opened a way to study the functions of miRNAs in SAR [113]. Some miRNAs like *miR472*, *miR482*, and *miR2109* suppress resistance (R) genes to regulate defense responses [128]. Although *miR846* and *miR825* have been reported to regulate Bacillus-induced ISR, they belong to non-conserved miRNAs, which exist only in some plant species [129]. *Bacillus velezensis* FZB42 can activate ISR to enhance plant defense response against pathogen infections. Two miRNAs, *miR169* and *miR395*, which were repressed in FZB42-treated plants especially, were selected as candidate ISR-associated miRNAs. All of them contained at least one cis-element associated with defense response, whereas the *miR169* family had the most abundant defense-related cis-elements [113]. In addition to the regulation of some abiotic stresses, the *miR169* family and its targets NF-YA genes participate in plant immunity [111]. This miRNA acts as a negative regulator in rice immunity against the blast fungus *Magnaporthe oryzae* by repressing the *NF-YAs* [130].

In summary, our results showed that some members in the *miR169* family (Fig 8A) might regulate NF-Y TFs to activate the ISR. This study is helpful in better understanding the regulatory roles of barley miRNAs in hormonal signaling and pathogen-activated ISR.

### Promoter analysis and potential regulatory elements associated with the pathogen resistance pathways

Promoters are regions of the genome that can bind TFs. The study of promoters, TFs, and downstream genes is a fascinating topic in post-genomics and can provide new insights into vital BPs [131, 132]. We performed this analysis to identify cis-acting elements located upstream of DEGs according to whether these are under common regulatory control. Eleven motifs with significant scores were identified (S8 Table).

Many cis-regulatory elements are responsive to pathogens and hormones. The most frequent cis-acting elements were highly matched to the MA1267.1 motif (Tables 5 and 6 and S7 Table), a C2H2 zinc finger motif (DOF5.8). These TFs play an important role in many BPs, e.g., flowering time, seed development, and responses to hormones and stresses [133]. He et al. (2015) [134] realized that the *Arabidopsis* DOF5.8 is an upstream regulator of a gene encoding an NAC family member in response to biotic and abiotic stresses [135]. More particularly, DOF5.8 was shown to play a significant role in the correct development of veins within the leaves of *A*. *thaliana*, and its promoter was demonstrated to be regulated by auxin [136]. Actually, the overexpression of this TF leading to a modification within the expression of the many genes, was involved in auxin biosynthesis, plant tissue formation, secondary cell wall deposition and similarly as signaling during physiological processes [137, 138].

Since cross-talk between plant hormone signaling pathways is critical for controlling the immune response during pathogen invasion, we investigate a basic helix-loop-helix (bHLH) transcription activator, which activates JA signaling in cereal when is localized to the nucleus [139]. The bHLHs are one of the largest TF families and participate in the regulation of plant growth and development [140, 141], iron homeostasis [142], root vascular cell proliferation [143], shoot branching [144], stomatal initiation [145], flowering time [146], pollen, gynoecium and fruit development [147], and grain yield [148]. bHLH is involved in the phytohormone-mediated pathogen stress adaptation and resistance development [149]. Following infection by fungal pathogens such as *Magnaporthe oryzae*, the SA signaling regulator, *NPR1* sequesters bHLH in the cytosol, which activates SA signaling but represses JA signaling. We, therefore, propose that bHLHs may regulate the SA/JA antagonism in cereals [139]. Further, wheat bHLH overexpression negatively regulated resistance to *Pseudomonas syringae* through JA and ET signaling in transgenic *Arabidopsis* [150]. Although some functions of bHLHs have been characterized, the biological functions of most of them remain unclear, especially in gramineous crops such as rice, maize, and wheat [149]. The subgroup of bHLHs (bHLH3, bHLH13, bHLH14, and bHLH17) in *Arabidopsis* was found to act as targets of JAZs and negatively regulate JA-mediated defense and development [151]. bHLHs are repressors that bind to cis-regulatory elements of MYC target genes and inhibit transcription [152]. In other words, stabilized JAZs and bHLH repressors counteract the activation of JA responses to fine-tune defense and development. Therefore, the accumulation of SA in response to biotrophs and insects interferes with JA defense gene expression via other routes [152].

### Co-expression network analysis and identification of hub genes

Co-expression network analysis of the DEGs was performed to discover processes and functions involved in response to pathogens and hormones (Fig 5 and S3 Table). Interestingly, 4 modules, including brown, blue, and turquoise for both datasets, were significantly related with response to defense pathways. After studying the genes within these modules, finally, four genes (*SSI2*, *PR1*, *PKT3*, and *LOX2*) related to biotic studies and two genes (*AOS* and *OPR3*) related to hormonal studies that were involved in the regulation of BPs were identified to further investigation (Fig 9 and S10 Table).

The *Arabidopsis ssi2* is a mutant line affected in both SA- and JA-mediated defense signaling. *SSI2* encodes a plastid-localized stearoyl-acyl carrier protein desaturase (SACPD) that desaturates stearic acid to oleic acid in the chloroplast. The *ssi2* line causes a truncation in the SSI2, which results in the loss of 90% of SACPD activity [153]. SACPD is an archetypical member of a family of soluble FAs desaturases; these enzymes play an important role in regulating the overall level of desaturated FAs in the cell and in modulating cross-talk among different defense signaling pathways [154]. The *ssi2* lines are stunted in size, show spontaneous death of the cell, constitutive overexpress of *PR1*, accumulation of high levels of SA, and exhibit enhanced resistance to pathogens. Taken together, *SSI2* appears to affect the activation of several defense responses by modulating a NIM1/NPR1-independent defense pathway. The double mutant lines of *ssi1 npr1-5* and *ssi2 npr1-5*, suppressed in constitutive SA signaling, show necrotic lesions and basal-level expression of *PR1* in the absence of a functional *NIM1/NPR1*. Therefore, an NPR1-dependent signaling pathway acts additively with the *ssi2*-induced NPR1-independent signaling pathway to enhance *PR1* expression [155]. In addition to regulating SAR, the *NIM1/NPR1* plays a role in ISR as well [156], which is activated in *Arabidopsis* after exposure of roots to certain nonpathogenic *Pseudomonas fluorescens* strains. Unlike SAR, induction of ISR does not require SA accumulation and is not related to the expression of SAR-associated *PRs*. However, like SAR, induction of ISR is dependent upon *NIM1/NPR1* [156]. Therefore, SAR and ISR appear to represent distinct defense pathways in Arabidopsis that are likely to differ in their modes of action, yet share a requirement for *NIM1/NPR1* [157]. How *NIM1/NPR1* modulates the expression of both SAR and ISR is unclear, but it is becoming apparent that *NIM1* plays a central role in multiple defense signaling pathways [158]. Because SACPD catalyzes a desaturation step required for JA biosynthesis [153], we attempted to determine whether the treatment of barley seedlings by MeJA can affect the *SSI2* expression (Fig 11A).

PR proteins, which are classified into 17 families, accumulate after pathogen infection or related situations in many plant species and cause defense responses, especially during the hypersensitive response (HR) [159]. Among the *PR* family, *PR1* has been frequently applied as a marker gene for SAR in many species [160]. One *PR1* homolog, *At2g14610*, encodes a basic protein that has been identified in many plant species as a marker of defense against oomycetes, fungi, bacteria, or viruses [161]. From these results, we tend to suppose that only *PR1* relates directly to pathogen resistance in *Arabidopsis* and the others contribute to other functions. In response to defense hormones, *PR1* was induced briefly by MeJA treatments (Fig 11B). Indeed, the MeJA treatment and pathogen infection induce the same set of *PRs*, suggesting JA/ET-signaling pathways are involved in pathogen resistance [162, 163].

Another hub gene is *PKT3* which encodes a 3-ketoacyl thiolase in peroxisomes that catalyzes the last step of the β-oxidation of fatty acids to produce one molecule of acetyl-CoA in each repeat cycle. This gene shows higher expression than the redundant homologs, such as *PKT1* and *PKT5*. It is now broadly accepted that *PKT3* acts in IAA biosynthesis through its β-oxidative decarboxylation in peroxisomes [164]. The coordinated induced expression of β-oxidation genes is essential to provide the energy supply for germination or post-development of germination. Mechanical damage triggers the local and systemic induction of *PKT3* in *Arabidopsis*. Although most of the β-oxidation genes were activated by wound-related factors such as dehydration and ABA, JA induces only *ACX1* and *PKT5* [101]. Reduced expression of *ACX1* or *PKT3* in transgenic plants, expressing their corresponding mRNAs in antisense orientation, was correlated with defective wound-activated synthesis of JA and reduced expression of JA-responsive genes. Induced expression of JA-responsive genes by exogenous application of JA was unaffected in those transgenic plants, suggesting that *ACX1* and *PKT3* play a major role in driving wound-activated responses by participating in the biosynthesis of JA [101].

Another hub gene is *AOS*, a key enzyme of the JA signaling pathway. The mutation of the single *AOS* in Arabidopsis leads to the complete elimination of JA production [81]. It has proved that the plants constitutively expressing AOS delay pathogen progression and exhibit more vigorous growth. They also showed the up-regulation of JA and SA-related genes, increased resistance to pathogens, and robust induction of *PRs*, such as *PR1*, *PR3*, and *PR5* [165, 166]. Taken together, these results demonstrate that the AOS is an important player in plant defense responses against pathogen infections and affects the JA biosynthetic pathway thus its expression in susceptible varieties may be a valuable tool to mitigate biotic stress responses [167].

Arabidopsis OPDA (12-oxophytodienoic acid) reductase (OPR3) catalyzes the reduction of OPDA to 3-oxo-2-(2′(Z)-pentenyl)-cyclopentane-1 octanoic acid (OPC-8:0). OPDA is known as a biosynthetic precursor of the JA. Although Arabidopsis contains at least two other *OPR* homologs, *OPR1* and *OPR2*, their protein products do not catalyze the reduction of OPDA [168]. Among the 6 wound-induced *OPRs* of *A*. *thaliana*, only *OPR3* is involved in JA biosynthesis [169]. Interestingly, inducing *OPR3* expression by JA indicates an opportunity for feedback regulation of gene expression. Expression of *opr3* in plants treated with OPDA differs from that in *opr3* lines treated with JA. In contrast to OPDA, JA induces the transcription of three homologous genes. Research results show that OPDA has different regulatory functions than JA and is an independent signaling molecule [170]. Generally, the plants are deficient in the biosynthesis of JA, and they accumulate OPDA when wounded. Significantly, *OPR3* has capable JA defense responses against insect pests and pathogens such as *Botrytis cinerea* and *Alternaria brassicicola*. Although topical application of JA and MeJA induces transcription of defense genes in Arabidopsis, OPDA alone is sufficient for these responses. This raises the question of which of these hormones represent the active signal molecules in plants [169]. The *OPR3* is the candidate hub gene that is validated for qPCR.

*Lipoxygenase* (*LOX*) is last hub gene candidate for more validation in qPCR. An enzyme that catalyzes the hydro-peroxidation of specific unsaturated fatty acids as well as initiates JA synthesis and converts linoleic acid into 13-hydroperoxy linolenic acid Therefore, *LOXs* are induced in the ISR pathway and involved in defense responses against the pathogen [171]. Among the 6 *LOXs* of Arabidopsis, four of them are *13-LOXs* (*LOX2*, *LOX3*, *LOX4*, *LOX6*), although their functions are only partly understood. It was thought that *LOX2* was involved in wound response for a long time [169]. The subsequent studies revealed that *LOX2* was responsible for the bulk of JA formation in pollen and stamen development in the first hours of wounding [172]. In mutants with defects in a JA-regulated signal transduction pathway, the regulation of JA-inducible genes might be altered. It has been reported that *A*. *thaliana LOX2* is induced by MeJA. *LOX2* is also involved in lipid peroxidation that occurs under abiotic and biotic stresses. A *LOX2* mediated double oxygenation of plastid galactolipids leading to the production of arabidopsides was observed upon pathogen infection, however it was not responsible for the pathogen-induced increase in JA [173]. Interestingly, the formation of lipid peroxides accompanies the synthesis of azelaic acid, a new signaling compound priming the immune responses [174].

### Validation of candidate genes

The results suggest that the gene expressions are reliable and mainly are consistent with those from data analysis. After MeJA treatment, as shown in Fig 11, the expression of *SSI2* was early induced. This gene causes biotic stress-induced constitutive SA signaling [153]. High levels of linolenic acid in *ssi2* mutant lines are required for the activation of certain ET/JA-mediated responses, because these lines are responsive to both of these hormones, but show normal levels of SA and *PRs* expression [153]. JA treatment would not be sufficient to activate *PDF1*.*2* expression in *ssi2* lines or restore resistance to pathogens since these mutants lack or have low levels of this co-activating signal [154]. Supporting this possibility is the discovery that the induction of *PR1* or injection of oleic acid into the leaves of these lines restores JA-inducible *PDF1*.*2* expression [175].

The gene expression analysis indicated that *PKT3* and *PR1* were induced to a significantly higher level in response to MeJA treatment (Figs 11 and S6). The induced *PR1* is a useful molecular marker for the SAR responses and is essential for the transduction of the SA signals [176]. The induction of the same set of *PRs* in response to pathogen infections, MeJA or ethephon, suggests the involvement of JA/ET-signaling pathways in mediating resistance against pathogens consistent with the potential hemibiotrophic nature. Indeed, the responses of the *PR1* homologs to defense signaling compounds indicate that the signaling pathways in rice conferred by SA and JA were synergistic as well as that by ET and JA, and SA and ET [160].

The induction of *LOX2*, *OPR3*, and *AOS* activates the defense responses against the pathogens and biosynthesis of JA, thus they can induce the ISR pathway [81]. These genes involved in JA biosynthesis were significantly induced during *Botrytis cinerea* infection in wild type, but their expression was reduced in the *coi1* mutant that is a critical component of the JA receptor [177]. Additionally, the activation of these genes was also repressed by SA, suggesting that the JA biosynthesis pathway may be a target for SA-mediated antagonism [178]. According to these results, based on global differential expression matrices, machine-learning approaches could help predict how potential plant elicitors act.

## Conclusions

Using barley transcriptome data, we integrated a meta-analysis method with gene co-expression network analysis. Based on these analyses, we identified genes that play a significant role against pathogens and in hormonal signaling. The results indicate that most of the DEGs influence a wide range of biological processes related to pathogen resistance and hormone signaling transduction. Because of the challenging environment, crops encountered numerous biotrophic and necrotrophic pathogens that required SA and JA signals to respond. The importance of TFs (e.g., WRKYs, MYBs, bHLHs, and bZIPs), kinases (e.g., RLK/Pelles), and miRNAs (e.g., miR6192) in the responses to pathogens was demonstrated and several novel interesting genes with unknown functions were identified. We showed that WHIRLY1 and NF-Y TF families, and miR169 family have a more important role in connection with SAR /ISR pathways and hormone-induced defenses. The results can be the center of more attention and study. The promoter analysis identified 11 overrepresented cis-acting elements such as zinc finger proteins in upstream regions of DEGs, which improve plant defense against fungal attack by ISR-dependent pathway. Moreover, it confirmed the results of the meta-analysis by showing the existence of pathogen-specific and consensus modules against pathogens and in hormonal signaling. The PPI network analysis helped to visualize and introduce the key genes such as *SSI2*, *PR1*, *PKT3*, *AOS*, *LOX2*, and *OPR3* related to hormonal signaling and pathogenic infections that were validated in qPCR. According to integrative data analysis, these genes showed a significant expression change after MeJA treatment in qPCR, the potential candidates for the regulation of JA biosynthesis. A number of the DEGs with high connectivity and conserved expression but with weak annotation were also recognized. It may contribute to a much better understanding of how barley responds to MeJA treatment resulting in ISR. Unlike SAR, induction of ISR does not require SA accumulation and is not related to the expression of SAR-associated *PRs*. However, like SAR, induction of ISR is dependent upon NIM1/NPR1. Thus, in addition to regulating SAR, NPR1/NIM1 has also been shown to be involved in the activation of ISR by the SSI2. We suggest these genes for more study, which might provide a more robust bio-signature for phenotypic traits as a more promising resource of molecular biomarkers and potential candidate genes for engineering pathogen resistance in barley.

## Supporting information

S1 Fig The barley seedlings in the glasshouse. The seedlings were treated with 100 μM MeJA (plus 0.1% Tween-20) in 0.1% ethanol. In addition, the controls were sprayed and watered with 0.1% Tween-20 in 0.1% ethanol. The leaf samples were collected at 3, 6, 24, and 48 hours after treatment.
(TIF)

S2 Fig Normalization process. Reducing heterogeneity among samples of studies for direct merging meta-analysis. (a) The E-GEOD-33398 study plot box related to biotic stresses in Affymetrix platform with 3 control samples and 3 treatment samples, was drawn in the pre-normalization stage. (b) The E-GEOD-33398 study plot box after normalization, where all comparisons that are not significant or are not equal to the change threshold are converted to a log 2 value to remove a possible error. This method ensured that weak expression fluctuations were more likely to be real biological signals than measurement errors or errors not corrected by RMA normalization. The biological errors and batch effects have been corrected. After preprocessing, the black lines of box plot are almost on the same straight line, indicating a high level of normalization. The horizontal axis stands for control and treatment different samples, while the vertical axis represents expression value. The black line in the box represents the expression median for each sample.
(TIF)

S3 Fig Weighted gene co-expression network analysis by dynamic tree cut method. (a) Shows a hierarchical cluster tree of the biotic DEGs. The branches and color bands represent the assigned module. The tips of the branches represent genes. (b) Shows a hierarchical cluster tree of the hormonal DEGs. The branches and color bands represent the assigned module. The tips of the branches represent genes. The color bands below the dendrogram show the cluster membership (identified modules) according to tree cut methods. The X-axis refers the results of the standard, constant height cut-off method at height 0.8. The Y-axis refers to the height of the dendrogram.
(TIF)

S4 Fig Venn diagrams of hub genes inferred from biotic and hormonal studies. Comparison of hub genes in biotic and hormonal studies identified 10 common genes.
(TIF)

S5 Fig The protein-protein interaction network using STRING database for candidate genes. (a) The network of interactions for *SSI2*, (b) *PR1*, (c) *PKT3* and (d) *AOS*, *OPR3* and *LOX2* are showed.
(TIF)

S6 Fig Comparative relative expression of the hub genes after 100 μM MeJA treatment. The different letters on the columns represent the significant differences given by Duncan’s multiple range statistical analysis. Vertical bars indicate ± SE of the mean (n = 3, *P-value* ≤ 0.05). The X-axis shows the times after MeJA treatment. As it is known, the target genes showed the highest expression at the time points of 6 h to 24 h and showed the lowest expression at the time of 48 h.
(TIF)

S1 Table Biotic/hormonal datasets retrieved from GEO and Array Express databases. (XLSX)

S2 Table List of identified DEGs by meta-analysis of biotic/hormonal datasets and their corresponding UniGene, UniProt, and TAIR IDs. (XLSX)

S3 Table Gene ontology of biotic and hormonal modules found by the DAVID database. (XLSX)

S4 Table List of top 30 hub genes in each module for biotic/hormonal datasets. (XLSX)

S5 Table List of transcription factors enriched for biotic/hormonal DEGs. (XLSX)

S6 Table List of the highest scoring miRNAs associated with biotic/hormonal DEGs that were identified using psRNA target server. (XLSX)

S7 Table List of known cis-acting elements biotic/hormonal DEGs by Tomtom search against the JASPAR database. (XLSX)

S8 Table Significant GO terms enriched by GOMO analysis for biotic/hormonal DEGs. (XLSX)

S9 Table Gene ontology consensus cis-acting elements of biotic/hormonal modules found by the DAVID database. (XLSX)

S10 Table The most relevant genes in biotic/hormonal datasets. (XLSX)

S1 Graphical abstract (TIF)

## Abbreviations

DEGs: Differentially Expressed Genes
TFs: Transcription Factors
PKT3: Peroxisomal 3-Ketoacyl-Coenzyme A Thiolase
PRs: Pathogenesis-Related genes
SSI2: Suppressor of Salicylic Acid-Insensitive 2
LOX2: Lipoxygenase 2
3OPR3: Oxophytodienoate-Reductase
AOS: Allene Oxide Synthase
MeJA: Methyl Jasmonic Acid
SA: Salicylic Acid
NPR1: Nonexpressor of Pathogenesis-Related Genes 1
SAR: Systemic Acquired Resistance
ISR: Induced Systemic Resistance
HR: Hypersensitive Reaction
PKs: Protein Kinases
WGCNA: Weighted Gene Co-expression Network Analysis

## References

[1] Ahmad M, Ali Q, Hafeez M, Malik A. Improvement for biotic and abiotic stress tolerance in crop plants. Biological and Clinical Sciences Research Journal. 2021;2021(1).

[2] Hasan MS, Singh V, Islam S, Islam MS, Ahsan R, Kaundal A, et al. Genome-wide identification and expression profiling of glutathione S-transferase family under multiple abiotic and biotic stresses in Medicago truncatula L. Plos one. 2021;16(2):e0247170. doi: 10.1371/journal.pone.0247170 33606812 PMC7894904

[3] Chausali N, Saxena J. Role of Bacillus Species in Alleviating Biotic Stress in Crops. Bacilli in Agrobiotechnology: Springer; 2022. p. 365–91.

[4] Arabi MIE, Al-Daoude A, Jawhar M, Shoaib EA-SA. Early events involved in barley response to phytopathogenic fungi with different lifestyles. MYCOPATH. 2021;18(1).

[5] Devanna B, Hosahatti R, Raghu S, Singh P, Jain P, Parameswaran C, et al. Advances in Genetics and Genomics for Management of Blast Disease in Cereal Crops. Blast Disease of Cereal Crops: Springer; 2021. p. 173–81.

[6] Sapkota R, Jørgensen LN, Boeglin L, Nicolaisen M. Fungal Communities of Spring Barley from Seedling Emergence to Harvest During a Severe Puccinia hordei Epidemic. Microbial Ecology. 2022:1–11. doi: 10.1007/s00248-022-01985-y 35229200

[7] Matušinsky P, Sedláková B, Bleša D. Compatible interaction of Brachypodium distachyon and endophytic fungus Microdochium bolleyi. PloS one. 2022;17(3):e0265357. doi: 10.1371/journal.pone.0265357 35286339 PMC8920291

[8] Tembo B, Mulenga RM, Sichilima S, M’siska KK, Mwale M, Chikoti PC, et al. Detection and characterization of fungus (Magnaporthe oryzae pathotype Triticum) causing wheat blast disease on rain-fed grown wheat (Triticum aestivum L.) in Zambia. PloS one. 2020;15(9):e0238724. 10.1371/journal.pone.0238724 PMC7505438 32956369

[9] Luo K, Yao X-J, Luo C, Hu X-S, Wang C-P, Wang Y, et al. Biological and morphological features associated with english grain aphid and bird cherry-oat aphid tolerance in winter wheat line XN98-10-35. Journal of Plant Growth Regulation. 2019;38(1):46–54.

[10] Chakraborty B, Chakraborty U. Molecular detection of fungal pathogens and induction of phytoimmunity using bioinoculants. Indian Phytopathology. 2021:1–16.

[11] Milne RJ, Dibley KE, Schnippenkoetter W, Mascher M, Lui AC, Wang L, et al. The wheat Lr67 gene from the sugar transport protein 13 family confers multipathogen resistance in barley. Plant Physiology. 2019;179(4):1285–97. doi: 10.1104/pp.18.00945 30305371 PMC6446772

[12] Wang J, Xu C, Sun Q, Xu J, Chai Y, Berg G, et al. Post-translational regulation of autophagy is involved in intra-microbiome suppression of fungal pathogens. Microbiome. 2021;9(1):1–18. 34092253 10.1186/s40168-021-01077-y PMC8182927

[13] Delp G, Gradin T, Åhman I, Jonsson LM. Microarray analysis of the interaction between the aphid Rhopalosiphum padi and host plants reveals both differences and similarities between susceptible and partially resistant barley lines. Molecular Genetics and Genomics. 2009;281(3):233–48. doi: 10.1007/s00438-008-0409-3 19085010

[14] Wang D, Amornsiripanitch N, Dong X. A genomic approach to identify regulatory nodes in the transcriptional network of systemic acquired resistance in plants. PLoS pathogens. 2006;2(11):e123. doi: 10.1371/journal.ppat.0020123 17096590 PMC1635530

[15] Kaul S, Koo HL, Jenkins J, Rizzo M, Rooney T, Tallon LJ, et al. Analysis of the genome sequence of the flowering plant Arabidopsis thaliana. nature. 2000;408(6814):796–815. doi: 10.1038/35048692 11130711

[16] Lu X, Huang W, Zhang S, Li F, Zhang H, Sun R, et al. Resistance Management through Brassica Crop–TuMV–Aphid Interactions: Retrospect and Prospects. Horticulturae. 2022;8(3):247.

[17] Bolhassani M, Niazi A, Tahmasebi A, Moghadam A. Identification of key genes associated with secondary metabolites biosynthesis by system network analysis in Valeriana officinalis. Journal of plant research. 2021;134(3):625–39. doi: 10.1007/s10265-021-01277-5 33829347

[18] Tahmasebi A, Ebrahimie E, Pakniyat H, Ebrahimi M, Mohammadi-Dehcheshmeh M. Tissue-specific transcriptional biomarkers in medicinal plants: Application of large-scale meta-analysis and computational systems biology. Gene. 2019;691:114–24. doi: 10.1016/j.gene.2018.12.056 30620887

[19] Joshi A, Rienks M, Theofilatos K, Mayr M. Systems biology in cardiovascular disease: a multiomics approach. Nature Reviews Cardiology. 2021;18(5):313–30. doi: 10.1038/s41569-020-00477-1 33340009

[20] Moghadam A, Niazi A, Afsharifar A, Taghavi SM. Expression of a recombinant anti-HIV and anti-tumor protein, MAP30, in nicotiana tobacum hairy roots: A pH-stable and thermophilic antimicrobial protein. PloS one. 2016;11(7):e0159653. 10.1371/journal.pone.0159653 PMC4961381 27459300

[21] Garosi C, Ferrante R, Vettori C, Paffetti D. Meta-Analysis as a Tool to Identify Candidate Genes Involved in the Fagus sylvatica L. Abiotic Stress Response. Forests. 2022;13(2):159.

[22] Johnson KR, Mallon BS, Fann YC, Chen KG. Multivariate meta-analysis reveals global transcriptomic signatures underlying distinct human naive-like pluripotent states. PloS one. 2021;16(5):e0251461. doi: 10.1371/journal.pone.0251461 33984026 PMC8118304

[23] Tahmasebi A, Niazi A. Comparison of Transcriptional Response of C 3 and C 4 Plants to Drought Stress Using Meta-Analysis and Systems Biology Approach. Frontiers in plant science. 2021;12:668736. doi: 10.3389/fpls.2021.668736 34276729 PMC8280774

[24] Winter C, Camarão AA, Steffen I, Jung K. Network meta-analysis of transcriptome expression changes in different manifestations of dengue virus infection. BMC genomics. 2022;23(1):1–15. 35220956 10.1186/s12864-022-08390-2 PMC8882220

[25] Pieterse CM, Leon-Reyes A, Van der Ent S, Van Wees SC. Networking by small-molecule hormones in plant immunity. Nature chemical biology. 2009;5(5):308–16. doi: 10.1038/nchembio.164 19377457

[26] Schulz P, Herde M, Romeis T. Calcium-dependent protein kinases: hubs in plant stress signaling and development. Plant physiology. 2013;163(2):523–30. doi: 10.1104/pp.113.222539 24014579 PMC3793034

[27] Torres MA, Jones JD, Dangl JL. Reactive oxygen species signaling in response to pathogens. Plant physiology. 2006;141(2):373–8. doi: 10.1104/pp.106.079467 16760490 PMC1475467

[28] Asai T, Tena G, Plotnikova J, Willmann MR, Chiu W-L, Gomez-Gomez L, et al. MAP kinase signalling cascade in Arabidopsis innate immunity. Nature. 2002;415(6875):977–83. doi: 10.1038/415977a 11875555

[29] Tao Y, Xie Z, Chen W, Glazebrook J, Chang H-S, Han B, et al. Quantitative nature of Arabidopsis responses during compatible and incompatible interactions with the bacterial pathogen Pseudomonas syringae. The Plant Cell. 2003;15(2):317–30. doi: 10.1105/tpc.007591 12566575 PMC141204

[30] Wise RP, Moscou MJ, Bogdanove AJ, Whitham SA. Transcript profiling in host–pathogen interactions. Annu Rev Phytopathol. 2007;45:329–69. doi: 10.1146/annurev.phyto.45.011107.143944 17480183

[31] Kazan K, Lyons R. Intervention of phytohormone pathways by pathogen effectors. The Plant Cell. 2014;26(6):2285–309. doi: 10.1105/tpc.114.125419 24920334 PMC4114936

[32] Haider S, Iqbal J, Naseer S, Shaukat M, Abbasi BA, Yaseen T, et al. Unfolding molecular switches in plant heat stress resistance: A comprehensive review. Plant Cell Reports. 2021:1–24. 10.1007/s00299-021-02754-w 34401950

[33] Kim M, Xi H, Park J. Genome-wide comparative analyses of GATA transcription factors among 19 Arabidopsis ecotype genomes: Intraspecific characteristics of GATA transcription factors. PloS one. 2021;16(5):e0252181. 10.1371/journal.pone.0252181 PMC8153473 34038437

[34] Wani SH, Anand S, Singh B, Bohra A, Joshi R. WRKY transcription factors and plant defense responses: latest discoveries and future prospects. Plant Cell Reports. 2021;40(7):1071–85. doi: 10.1007/s00299-021-02691-8 33860345

[35] Sukumari Nath V, Kumar Mishra A, Kumar A, Matoušek J, Jakše J. Revisiting the role of transcription factors in coordinating the defense response against citrus bark cracking viroid infection in commercial hop (Humulus Lupulus L.). Viruses. 2019;11(5):419. doi: 10.3390/v11050419 31060295 PMC6563305

[36] Dey S, Wenig M, Langen G, Sharma S, Kugler KG, Knappe C, et al. Bacteria-triggered systemic immunity in barley is associated with WRKY and ETHYLENE RESPONSIVE FACTORs but not with salicylic acid. Plant physiology. 2014;166(4):2133–51. doi: 10.1104/pp.114.249276 25332505 PMC4256861

[37] Pieterse CM, Zamioudis C, Berendsen RL, Weller DM, Van Wees SC, Bakker PA. Induced systemic resistance by beneficial microbes. Annual review of phytopathology. 2014;52:347–75. doi: 10.1146/annurev-phyto-082712-102340 24906124

[38] Walters DR, Ratsep J, Havis ND. Controlling crop diseases using induced resistance: challenges for the future. Journal of experimental botany. 2013;64(5):1263–80. doi: 10.1093/jxb/ert026 23386685

[39] Vlot AC, Sales JH, Lenk M, Bauer K, Brambilla A, Sommer A, et al. Systemic propagation of immunity in plants. New Phytologist. 2021;229(3):1234–50. doi: 10.1111/nph.16953 32978988

[40] Rodriguez PA, Rothballer M, Chowdhury SP, Nussbaumer T, Gutjahr C, Falter-Braun P. Systems biology of plant-microbiome interactions. Molecular plant. 2019;12(6):804–21. doi: 10.1016/j.molp.2019.05.006 31128275

[41] Bernsdorff F, Döring A-C, Gruner K, Schuck S, Bräutigam A, Zeier J. Pipecolic acid orchestrates plant systemic acquired resistance and defense priming via salicylic acid-dependent and-independent pathways. The Plant Cell. 2016;28(1):102–29. doi: 10.1105/tpc.15.00496 26672068 PMC4746677

[42] Venegas-Molina J, Proietti S, Pollier J, Orozco-Freire W, Ramirez-Villacis D, Leon-Reyes A. Induced tolerance to abiotic and biotic stresses of broccoli and Arabidopsis after treatment with elicitor molecules. Scientific reports. 2020;10(1):1–17. 32587286 10.1038/s41598-020-67074-7 PMC7316721

[43] Betsuyaku S, Katou S, Takebayashi Y, Sakakibara H, Nomura N, Fukuda H. Salicylic acid and jasmonic acid pathways are activated in spatially different domains around the infection site during effector-triggered immunity in Arabidopsis thaliana. Plant and Cell Physiology. 2018;59(1):8–16. doi: 10.1093/pcp/pcx181 29177423 PMC6012717

[44] Zhao S, Li Y. Current understanding of the interplays between host hormones and plant viral infections. PLoS Pathogens. 2021;17(2):e1009242. doi: 10.1371/journal.ppat.1009242 33630970 PMC7906326

[45] Saleem M, Fariduddin Q, Castroverde CDM. Salicylic acid: A key regulator of redox signalling and plant immunity. Plant Physiology and Biochemistry. 2021;168:381–97. doi: 10.1016/j.plaphy.2021.10.011 34715564

[46] Raffaele S, Rivas S, Roby D. An essential role for salicylic acid in AtMYB30-mediated control of the hypersensitive cell death program in Arabidopsis. FEBS letters. 2006;580(14):3498–504. doi: 10.1016/j.febslet.2006.05.027 16730712

[47] Mo S, Zhang Y, Wang X, Yang J, Sun Z, Zhang D, et al. Cotton GhSSI2 isoforms from the stearoyl acyl carrier protein fatty acid desaturase family regulate Verticillium wilt resistance. Molecular plant pathology. 2021;22(9):1041–56. doi: 10.1111/mpp.13093 34169624 PMC8358998

[48] Osório J. Landscape and mechanisms of transcription factor cooperativity. Nature Reviews Genetics. 2016;17(1):5-. 10.1038/nrg.2015.11 26593418

[49] Krishnatreya DB, Agarwala N, Gill SS, Bandyopadhyay T. Understanding the role of miRNAs for improvement of tea quality and stress tolerance. Journal of Biotechnology. 2021;328:34–46. doi: 10.1016/j.jbiotec.2020.12.019 33421509

[50] Yu Q, Jin X, Liu C, Wen Y. An Integrated Analysis of Transcriptome and miRNA Sequencing Provides Insights into the Dynamic Regulations during Flower Morphogenesis in Petunia. Horticulturae. 2022;8(4):284.

[51] Puentes A, Zhao T, Lundborg L, Björklund N, Borg-Karlson A-K. Variation in Methyl Jasmonate-Induced Defense Among Norway Spruce Clones and Trade-Offs in Resistance Against a Fungal and an Insect Pest. Frontiers in plant science. 2021:962. 10.3389/fpls.2021.678959 PMC8182065 34108985

[52] Ding L-N, Yang G-X, Yang R-Y, Cao J, Zhou Y. Investigating interactions of salicylic acid and jasmonic acid signaling pathways in monocots wheat. Physiological and Molecular Plant Pathology. 2016;93:67–74.

[53] Nahar K, Kyndt T, Nzogela YB, Gheysen G. Abscisic acid interacts antagonistically with classical defense pathways in rice–migratory nematode interaction. New Phytologist. 2012;196(3):901–13. doi: 10.1111/j.1469-8137.2012.04310.x 22985247

[54] Brazma A, Hingamp P, Quackenbush J, Sherlock G, Spellman P, Stoeckert C, et al. Minimum information about a microarray experiment (MIAME)—toward standards for microarray data. Nature genetics. 2001;29(4):365–71. doi: 10.1038/ng1201-365 11726920

[55] Cohen SP, Leach JE. Abiotic and biotic stresses induce a core transcriptome response in rice. Scientific reports. 2019;9(1):1–11. 31000746 10.1038/s41598-019-42731-8 PMC6472405

[56] Irizarry RA, Bolstad BM, Collin F, Cope LM, Hobbs B, Speed TP. Summaries of Affymetrix GeneChip probe level data. Nucleic acids research. 2003;31(4):e15-e. doi: 10.1093/nar/gng015 12582260 PMC150247

[57] Durinck S. Pre-processing of microarray data and analysis of differential expression. Bioinformatics: Springer; 2008. p. 89–110. 10.1007/978-1-60327-159-2_4 18563370

[58] Ritchie ME, Silver J, Oshlack A, Holmes M, Diyagama D, Holloway A, et al. A comparison of background correction methods for two-colour microarrays. Bioinformatics. 2007;23(20):2700–7. doi: 10.1093/bioinformatics/btm412 17720982

[59] Bolstad BM, Irizarry RA, Åstrand M, Speed TP. A comparison of normalization methods for high density oligonucleotide array data based on variance and bias. Bioinformatics. 2003;19(2):185–93. doi: 10.1093/bioinformatics/19.2.185 12538238

[60] Smyth GK. Limma: linear models for microarray data. Bioinformatics and computational biology solutions using R and Bioconductor: Springer; 2005. p. 397–420.

[61] Hong F, Breitling R. A comparison of meta-analysis methods for detecting differentially expressed genes in microarray experiments. Bioinformatics. 2008;24(3):374–82. doi: 10.1093/bioinformatics/btm620 18204063

[62] Balan B, Caruso T, Martinelli F. Gaining insight into exclusive and common transcriptomic features linked with biotic stress responses in Malus. Frontiers in plant science. 2017;8:1569. doi: 10.3389/fpls.2017.01569 28955361 PMC5601412

[63] Du Z, Zhou X, Ling Y, Zhang Z, Su Z. agriGO: a GO analysis toolkit for the agricultural community. Nucleic acids research. 2010;38(suppl_2):W64–W70. doi: 10.1093/nar/gkq310 20435677 PMC2896167

[64] Moghadam A, Tahmasebi A, Taghizadeh MS, Bolhassani M, Ebrahimie E. Identification of LncRNAs involved in secondary metabolites biosynthesis in Echinacea purpurea. Scientific reports. 2022. doi: 0f8b2830-99d8-452c-ab86-53c38c902621.

[65] Shaar-Moshe L, Hübner S, Peleg Z. Identification of conserved drought-adaptive genes using a cross-species meta-analysis approach. BMC plant biology. 2015;15(1):111. doi: 10.1186/s12870-015-0493-6 25935420 PMC4417316

[66] Zheng Y, Jiao C, Sun H, Rosli HG, Pombo MA, Zhang P, et al. iTAK: a program for genome-wide prediction and classification of plant transcription factors, transcriptional regulators, and protein kinases. Molecular plant. 2016;9(12):1667–70. 27717919 10.1016/j.molp.2016.09.014

[67] Dai X, Zhuang Z, Zhao PX. psRNATarget: a plant small RNA target analysis server (2017 release). Nucleic acids research. 2018;46(W1):W49–W54. doi: 10.1093/nar/gky316 29718424 PMC6030838

[68] Ye J, Zhang Y, Cui H, Liu J, Wu Y, Cheng Y, et al. WEGO 2.0: a web tool for analyzing and plotting GO annotations, 2018 update. Nucleic acids research. 2018;46(W1):W71–W5. doi: 10.1093/nar/gky400 29788377 PMC6030983

[69] Montenegro JD. Gene Co-expression Network Analysis. Plant Bioinformatics: Springer; 2022. p. 387–404. 10.1007/978-1-0716-2067-0_19 35037216

[70] Amiri F, Moghadam A, Tahmasebi A, Niazi A. Identification of key genes involved in secondary metabolite biosynthesis in Digitalis purpurea. PLOS One. 2022. 10.1371/journal.pone.0277293 PMC9997893 36893121

[71] Wang J, Fan Y, Mao L, Qu C, Lu K, Li J, et al. Genome-wide association study and transcriptome analysis dissect the genetic control of silique length in Brassica napus L. Biotechnology for biofuels. 2021;14(1):1–14. 34743746 10.1186/s13068-021-02064-z PMC8573943

[72] Li CY, Cai J-H, Tsai JJ, Wang CC. Identification of hub genes associated with development of head and neck squamous cell carcinoma by integrated bioinformatics analysis. Frontiers in Oncology. 2020;10:681. doi: 10.3389/fonc.2020.00681 32528874 PMC7258718

[73] Moghadam A, Foroozan E, Tahmasebi A, Taghizadeh MS, Bolhassani M, Jafari M. System network analysis of Rosmarinus officinalis transcriptome ─ key genes in biosynthesis of secondary metabolites. PLOS One. 2022. 10.1371/journal.pone.0282316 PMC9980811 36862714

[74] Moghadam A, Taghavi S, Niazi A, Djavaheri M, Ebrahimie E. Isolation and in silico functional analysis of MtATP6, a 6-kDa subunit of mitochondrial F. Genetics and Molecular Research. 2012;11(4):3547–67. 23096681 10.4238/2012.October.4.3

[75] Shahriari AG, Soltani Z, Tahmasebi A, Poczai P. Integrative System Biology Analysis of Transcriptomic Responses to Drought Stress in Soybean (Glycine max L.). Genes. 2022;13(10):1732. doi: 10.3390/genes13101732 36292617 PMC9602024

[76] Bailey TL, Boden M, Buske FA, Frith M, Grant CE, Clementi L, et al. MEME SUITE: tools for motif discovery and searching. Nucleic acids research. 2009;37(suppl_2):W202–W8. doi: 10.1093/nar/gkp335 19458158 PMC2703892

[77] Gupta S, Stamatoyannopoulos JA, Bailey TL, Noble WS. Quantifying similarity between motifs. Genome biology. 2007;8(2):1–9. doi: 10.1186/gb-2007-8-2-r24 17324271 PMC1852410

[78] Khan A, Fornes O, Stigliani A, Gheorghe M, Castro-Mondragon JA, Van Der Lee R, et al. JASPAR 2018: update of the open-access database of transcription factor binding profiles and its web framework. Nucleic acids research. 2018;46(D1):D260–D6. doi: 10.1093/nar/gkx1126 29140473 PMC5753243

[79] Buske FA, Bodén M, Bauer DC, Bailey TL. Assigning roles to DNA regulatory motifs using comparative genomics. Bioinformatics. 2010;26(7):860–6. doi: 10.1093/bioinformatics/btq049 20147307 PMC2844991

[80] Wang M, Wang L, Pu L, Li K, Feng T, Zheng P, et al. LncRNAs related key pathways and genes in ischemic stroke by weighted gene co-expression network analysis (WGCNA). Genomics. 2020;112(3):2302–8. doi: 10.1016/j.ygeno.2020.01.001 31923616

[81] Leon-Reyes A, Van der Does D, De Lange ES, Delker C, Wasternack C, Van Wees S, et al. Salicylate-mediated suppression of jasmonate-responsive gene expression in Arabidopsis is targeted downstream of the jasmonate biosynthesis pathway. Planta. 2010;232(6):1423–32. doi: 10.1007/s00425-010-1265-z 20839007 PMC2957573

[82] Yang W, Dong R, Liu L, Hu Z, Li J, Wang Y, et al. A novel mutant allele of SSI2 confers a better balance between disease resistance and plant growth inhibition on Arabidopsis thaliana. BMC Plant Biology. 2016;16(1):1–13. doi: 10.1186/s12870-016-0898-x 27669891 PMC5037883

[83] Liu XQ, Bai XQ, Qian Q, Wang XJ, Chen MS, Chu CC. OsWRKY03, a rice transcriptional activator that functions in defense signaling pathway upstream of OsNPR1. Cell research. 2005;15(8):593–603. doi: 10.1038/sj.cr.7290329 16117849

[84] Hernández ML, Whitehead L, He Z, Gazda V, Gilday A, Kozhevnikova E, et al. A cytosolic acyltransferase contributes to triacylglycerol synthesis in sucrose-rescued Arabidopsis seed oil catabolism mutants. Plant Physiology. 2012;160(1):215–25. doi: 10.1104/pp.112.201541 22760209 PMC3440200

[85] Kunz H-H, Scharnewski M, Feussner K, Feussner I, Flügge U-I, Fulda M, et al. The ABC transporter PXA1 and peroxisomal β-oxidation are vital for metabolism in mature leaves of Arabidopsis during extended darkness. The Plant Cell. 2009;21(9):2733–49. 19794119 10.1105/tpc.108.064857 PMC2768912

[86] Moghadam AA, Ebrahimie E, Taghavi SM, Niazi A, Babgohari MZ, Deihimi T, et al. How the nucleus and mitochondria communicate in energy production during stress: nuclear MtATP6, an early-stress responsive gene, regulates the mitochondrial F1F0-ATP synthase complex. Molecular biotechnology. 2013;54(3):756–69. 23208548 10.1007/s12033-012-9624-6

[87] Mhiri W, Ceylan M, Turgut-Kara N, Nalbantoğlu B, Çakır Ö. Transcriptomic analysis reveals responses to Cycloastragenol in Arabidopsis thaliana. PloS one. 2020;15(12):e0242986. doi: 10.1371/journal.pone.0242986 33301486 PMC7728452

[88] Zhao K, Kong D, Jin B, Smolke CD, Rhee SY. A novel bivalent chromatin associates with rapid induction of camalexin biosynthesis genes in response to a pathogen signal in Arabidopsis. Elife. 2021;10:e69508. doi: 10.7554/eLife.69508 34523419 PMC8547951

[89] Andersen EJ, Ali S, Byamukama E, Yen Y, Nepal MP. Disease resistance mechanisms in plants. Genes. 2018;9(7):339. doi: 10.3390/genes9070339 29973557 PMC6071103

[90] Gupta R, Leibman‐Markus M, Pizarro L, Bar M. Cytokinin induces bacterial pathogen resistance in tomato. Plant Pathology. 2021;70(2):318–25.

[91] Ho HL. Functional roles of plant protein kinases in signal transduction pathways during abiotic and biotic stress. J Biodivers Bioprospect Dev. 2015;2:147.

[92] He L, Li M, Qiu Z, Chen D, Zhang G, Wang X, et al. Primary leaf‐type ferredoxin 1 participates in photosynthetic electron transport and carbon assimilation in rice. The Plant Journal. 2020;104(1):44–58. 32603511 10.1111/tpj.14904

[93] Zhang Y, Li X. Salicylic acid: biosynthesis, perception, and contributions to plant immunity. Current opinion in plant biology. 2019;50:29–36. doi: 10.1016/j.pbi.2019.02.004 30901692

[94] Pascual MB, El-Azaz J, de la Torre FN, Cañas RA, Avila C, Cánovas FM. Biosynthesis and metabolic fate of phenylalanine in conifers. Frontiers in plant science. 2016;7:1030. doi: 10.3389/fpls.2016.01030 27468292 PMC4942462

[95] Martinelli F, Uratsu SL, Albrecht U, Reagan RL, Phu ML, Britton M, et al. Transcriptome profiling of citrus fruit response to huanglongbing disease. PloS one. 2012;7(5):e38039. doi: 10.1371/journal.pone.0038039 22675433 PMC3364978

[96] Falchi R, Bonghi C, Drincovich MF, Famiani F, Lara MV, Walker RP, et al. Sugar metabolism in stone fruit: source-sink relationships and environmental and agronomical effects. Frontiers in Plant Science. 2020;11:1820. doi: 10.3389/fpls.2020.573982 33281843 PMC7691294

[97] Punelli F, Al Hassan M, Fileccia V, Uva P, Pasquini G, Martinelli F. A microarray analysis highlights the role of tetrapyrrole pathways in grapevine responses to “stolbur” phytoplasma, phloem virus infections and recovered status. Physiological and Molecular Plant Pathology. 2016;93:129–37.

[98] Zhou M, Wang W. Recent advances in synthetic chemical inducers of plant immunity. Frontiers in plant science. 2018:1613. doi: 10.3389/fpls.2018.01613 30459795 PMC6232518

[99] Manners JM, Penninckx IA, Vermaere K, Kazan K, Brown RL, Morgan A, et al. The promoter of the plant defensin gene PDF1. 2 from Arabidopsis is systemically activated by fungal pathogens and responds to methyl jasmonate but not to salicylic acid. Plant molecular biology. 1998;38(6):1071–80. doi: 10.1023/a:1006070413843 9869413

[100] Zhang N, Zhou S, Yang D, Fan Z. Revealing shared and distinct genes responding to JA and SA signaling in Arabidopsis by meta-analysis. Frontiers in plant science. 2020;11:908. doi: 10.3389/fpls.2020.00908 32670328 PMC7333171

[101] Castillo MC, Martínez C, Buchala A, Métraux J-P, León J. Gene-specific involvement of β-oxidation in wound-activated responses in Arabidopsis. Plant physiology. 2004;135(1):85–94. 15141068 10.1104/pp.104.039925 PMC429335

[102] Mosblech A, Feussner I, Heilmann I. Oxylipins: structurally diverse metabolites from fatty acid oxidation. Plant Physiology and Biochemistry. 2009;47(6):511–7. doi: 10.1016/j.plaphy.2008.12.011 19167233

[103] Spoel SH, Koornneef A, Claessens SM, Korzelius JP, Van Pelt JA, Mueller MJ, et al. NPR1 modulates cross-talk between salicylate-and jasmonate-dependent defense pathways through a novel function in the cytosol. The Plant Cell. 2003;15(3):760–70. doi: 10.1105/tpc.009159 12615947 PMC150028

[104] Wasternack C. Jasmonates: an update on biosynthesis, signal transduction and action in plant stress response, growth and development. Annals of botany. 2007;100(4):681–97. doi: 10.1093/aob/mcm079 17513307 PMC2749622

[105] Balan B, Marra FP, Caruso T, Martinelli F. Transcriptomic responses to biotic stresses in Malus x domestica: a meta-analysis study. Scientific reports. 2018;8(1):1–12. 29386527 10.1038/s41598-018-19348-4 PMC5792587

[106] Tahmasebi A, Ashrafi-Dehkordi E, Shahriari AG, Mazloomi SM, Ebrahimie E. Integrative meta-analysis of transcriptomic responses to abiotic stress in cotton. Progress in biophysics and molecular biology. 2019;146:112–22. doi: 10.1016/j.pbiomolbio.2019.02.005 30802474

[107] Krupinska K, Dähnhardt D, Fischer-Kilbienski I, Kucharewicz W, Scharrenberg C, Trösch M, et al. Identification of WHIRLY1 as a factor binding to the promoter of the stress-and senescence-associated gene HvS40. Journal of plant growth regulation. 2014;33(1):91–105.

[108] Lin W, Zhang H, Huang D, Schenke D, Cai D, Wu B, et al. Dual-located WHIRLY1 affects salicylic acid homeostasis via coordination of ICS1, PAL1 and BSMT1 during Arabidopsis plant aging. bioRxiv. 2020.

[109] Krupinska K, Haussühl K, Schäfer A, van der Kooij TA, Leckband G, Lörz H, et al. A novel nucleus-targeted protein is expressed in barley leaves during senescence and pathogen infection. Plant physiology. 2002;130(3):1172–80. doi: 10.1104/pp.008565 12427984 PMC166638

[110] Desveaux D, Subramaniam R, Després C, Mess J-N, Lévesque C, Fobert PR, et al. A “Whirly” transcription factor is required for salicylic acid-dependent disease resistance in Arabidopsis. Developmental cell. 2004;6(2):229–40. doi: 10.1016/s1534-5807(04)00028-0 14960277

[111] Hanemian M, Barlet X, Sorin C, Yadeta KA, Keller H, Favery B, et al. Arabidopsis CLAVATA 1 and CLAVATA 2 receptors contribute to Ralstonia solanacearum pathogenicity through a miR169‐dependent pathway. New Phytologist. 2016;211(2):502–15. 26990325 10.1111/nph.13913

[112] Alam MM, Tanaka T, Nakamura H, Ichikawa H, Kobayashi K, Yaeno T, et al. Overexpression of a rice heme activator protein gene (Os HAP 2E) confers resistance to pathogens, salinity and drought, and increases photosynthesis and tiller number. Plant Biotechnology Journal. 2015;13(1):85–96. doi: 10.1111/pbi.12239 25168932

[113] Xie S, Yu H, Li E, Wang Y, Liu J, Jiang H. Identification of miRNAs involved in Bacillus velezensis FZB42-activated induced systemic resistance in maize. International journal of molecular sciences. 2019;20(20):5057. doi: 10.3390/ijms20205057 31614702 PMC6829523

[114] Cai Q, Wang J-J, Xie J-T, Jiang D-H, Keyhani NO. The Spt10 GNAT Superfamily Protein Modulates Development, Cell Cycle Progression and Virulence in the Fungal Insect Pathogen, Beauveria bassiana. Journal of Fungi. 2021;7(11):905. doi: 10.3390/jof7110905 34829192 PMC8619123

[115] Garcýa-Laynes S, Herrera-Valencia VA, Tamayo-Torres LG, Limones-Briones V, Barredo-Pool FA, Baas-Espinola FM, et al. The Banana MaWRKY18, MaWRKY45, MaWRKY60 and MaWRKY70 Genes Encode Functional Transcription Factors and Display Differential Expression in Response to Defense Phytohormones. Genes. 2022;13(10):1891. doi: 10.3390/genes13101891 36292777 PMC9602068

[116] Liu X, Yang S, Yu C-W, Chen C-Y, Wu K. Histone acetylation and plant development. The Enzymes. 2016;40:173–99. doi: 10.1016/bs.enz.2016.08.001 27776781

[117] Xue-Franzén Y, Henriksson J, Bürglin TR, Wright AP. Distinct roles of the Gcn5 histone acetyltransferase revealed during transient stress-induced reprogramming of the genome. BMC genomics. 2013;14(1):1–15. doi: 10.1186/1471-2164-14-479 23865462 PMC3723427

[118] Birkenbihl RP, Liu S, Somssich IE. Transcriptional events defining plant immune responses. Current Opinion in Plant Biology. 2017;38:1–9. doi: 10.1016/j.pbi.2017.04.004 28458046

[119] Ye Y, Ding Y, Jiang Q, Wang F, Sun J, Zhu C. The role of receptor-like protein kinases (RLKs) in abiotic stress response in plants. Plant cell reports. 2017;36(2):235–42. doi: 10.1007/s00299-016-2084-x 27933379

[120] Lehti-Shiu MD, Zou C, Hanada K, Shiu S-H. Evolutionary history and stress regulation of plant receptor-like kinase/pelle genes. Plant Physiology. 2009;150(1):12–26. doi: 10.1104/pp.108.134353 19321712 PMC2675737

[121] Acharya BR, Raina S, Maqbool SB, Jagadeeswaran G, Mosher SL, Appel HM, et al. Overexpression of CRK13, an Arabidopsis cysteine‐rich receptor‐like kinase, results in enhanced resistance to Pseudomonas syringae. The Plant Journal. 2007;50(3):488–99. doi: 10.1111/j.1365-313X.2007.03064.x 17419849

[122] Miya A, Albert P, Shinya T, Desaki Y, Ichimura K, Shirasu K, et al. CERK1, a LysM receptor kinase, is essential for chitin elicitor signaling in Arabidopsis. Proceedings of the National Academy of Sciences. 2007;104(49):19613–8. doi: 10.1073/pnas.0705147104 18042724 PMC2148337

[123] Dong X. Genetic dissection of systemic acquired resistance. Current opinion in plant biology. 2001;4(4):309–14. doi: 10.1016/s1369-5266(00)00178-3 11418340

[124] Djami-Tchatchou AT, Sanan-Mishra N, Ntushelo K, Dubery IA. Functional roles of microRNAs in agronomically important plants—potential as targets for crop improvement and protection. Frontiers in plant science. 2017;8:378. doi: 10.3389/fpls.2017.00378 28382044 PMC5360763

[125] Anjali NN, Sabu KK. Role of miRNAs in Abiotic and Biotic Stress Management in Crop Plants. Sustainable Agriculture in the Era of Climate Change: Springer; 2020. p. 513–32.

[126] Lv S, Nie X, Wang L, Du X, Biradar SS, Jia X, et al. Identification and characterization of microRNAs from barley (Hordeum vulgare L.) by high-throughput sequencing. International journal of molecular sciences. 2012;13(3):2973–84. doi: 10.3390/ijms13032973 22489137 PMC3317698

[127] Curaba J, Spriggs A, Taylor J, Li Z, Helliwell C. miRNA regulation in the early development of barley seed. BMC plant biology. 2012;12(1):1–16. doi: 10.1186/1471-2229-12-120 22838835 PMC3443071

[128] Boccara M, Sarazin A, Thiebeauld O, Jay F, Voinnet O, Navarro L, et al. The Arabidopsis miR472-RDR6 silencing pathway modulates PAMP-and effector-triggered immunity through the post-transcriptional control of disease resistance genes. PLoS pathogens. 2014;10(1):e1003883. 10.1371/journal.ppat.1003883 PMC3894208 24453975

[129] Xie S, Jiang H, Ding T, Xu Q, Chai W, Cheng B. Bacillus amyloliquefaciens FZB42 represses plant miR846 to induce systemic resistance via a jasmonic acid‐dependent signalling pathway. Molecular plant pathology. 2018;19(7):1612–23. doi: 10.1111/mpp.12634 29090851 PMC6638179

[130] Li Y, Zhao S-L, Li J-L, Hu X-H, Wang H, Cao X-L, et al. Osa-miR169 negatively regulates rice immunity against the blast fungus Magnaporthe oryzae. Frontiers in plant science. 2017;8:2. doi: 10.3389/fpls.2017.00002 28144248 PMC5239796

[131] Lemon B, Tjian R. Orchestrated response: a symphony of transcription factors for gene control. Genes & development. 2000;14(20):2551–69. doi: 10.1101/gad.831000 11040209

[132] Sandelin A, Alkema W, Engström P, Wasserman WW, Lenhard B. JASPAR: an open‐access database for eukaryotic transcription factor binding profiles. Nucleic acids research. 2004;32(suppl_1):D91–D4. doi: 10.1093/nar/gkh012 14681366 PMC308747

[133] Ruta V, Longo C, Lepri A, De Angelis V, Occhigrossi S, Costantino P, et al. The DOF transcription factors in seed and seedling development. Plants. 2020;9(2):218. doi: 10.3390/plants9020218 32046332 PMC7076670

[134] He L, Su C, Wang Y, Wei Z. ATDOF5. 8 protein is the upstream regulator of ANAC069 and is responsive to abiotic stress. Biochimie. 2015;110:17–24. doi: 10.1016/j.biochi.2014.12.017 25572919

[135] Klees S, Lange TM, Bertram H, Rajavel A, Schlüter J-S, Lu K, et al. In Silico Identification of the Complex Interplay between Regulatory SNPs, Transcription Factors, and Their Related Genes in Brassica napus L. Using Multi-Omics Data. International Journal of Molecular Sciences. 2021;22(2):789. doi: 10.3390/ijms22020789 33466789 PMC7830561

[136] Konishi M, Donner TJ, Scarpella E, Yanagisawa S. MONOPTEROS directly activates the auxin-inducible promoter of the Dof5. 8 transcription factor gene in Arabidopsis thaliana leaf provascular cells. Journal of experimental botany. 2015;66(1):283–91. doi: 10.1093/jxb/eru418 25336688 PMC4265163

[137] Guerriero G, Piasecki E, Berni R, Xu X, Legay S, Hausman J-F. Identification of Callose Synthases in Stinging Nettle and Analysis of Their Expression in Different Tissues. International journal of molecular sciences. 2020;21(11):3853. doi: 10.3390/ijms21113853 32481765 PMC7313033

[138] Konishi M, Yanagisawa S. Transcriptional repression caused by Dof5. 8 is involved in proper vein network formation in Arabidopsis thaliana leaves. Journal of plant research. 2015;128(4):643–52. doi: 10.1007/s10265-015-0712-0 25794540

[139] Meng F, Yang C, Cao J, Chen H, Pang J, Zhao Q, et al. A bHLH transcription activator regulates defense signaling by nucleo‐cytosolic trafficking in rice. Journal of Integrative Plant Biology. 2020;62(10):1552–73. doi: 10.1111/jipb.12922 32129570

[140] Gangappa SN, Kumar SV. DET1 and HY5 control PIF4-mediated thermosensory elongation growth through distinct mechanisms. Cell reports. 2017;18(2):344–51. doi: 10.1016/j.celrep.2016.12.046 28076780 PMC5263232

[141] Li K, Yu R, Fan L-M, Wei N, Chen H, Deng XW. DELLA-mediated PIF degradation contributes to coordination of light and gibberellin signalling in Arabidopsis. Nature communications. 2016;7(1):1–11. doi: 10.1038/ncomms11868 27282989 PMC4906400

[142] Li X, Zhang H, Ai Q, Liang G, Yu D. Two bHLH transcription factors, bHLH34 and bHLH104, regulate iron homeostasis in Arabidopsis thaliana. Plant Physiology. 2016;170(4):2478–93. doi: 10.1104/pp.15.01827 26921305 PMC4825117

[143] Ohashi-Ito K, Fukuda H. Functional mechanism of bHLH complexes during early vascular development. Current opinion in plant biology. 2016;33:42–7. doi: 10.1016/j.pbi.2016.06.003 27314622

[144] Komatsu K, Maekawa M, Ujiie S, Satake Y, Furutani I, Okamoto H, et al. LAX and SPA: major regulators of shoot branching in rice. Proceedings of the National Academy of Sciences. 2003;100(20):11765–70. doi: 10.1073/pnas.1932414100 13130077 PMC208832

[145] Raissig MT, Abrash E, Bettadapur A, Vogel JP, Bergmann DC. Grasses use an alternatively wired bHLH transcription factor network to establish stomatal identity. Proceedings of the National Academy of Sciences. 2016;113(29):8326–31. doi: 10.1073/pnas.1606728113 27382177 PMC4961163

[146] Ito S, Song YH, Josephson-Day AR, Miller RJ, Breton G, Olmstead RG, et al. FLOWERING BHLH transcriptional activators control expression of the photoperiodic flowering regulator CONSTANS in Arabidopsis. Proceedings of the National Academy of Sciences. 2012;109(9):3582–7. doi: 10.1073/pnas.1118876109 22334645 PMC3295255

[147] Ko S-S, Li M-J, Sun-Ben Ku M, Ho Y-C, Lin Y-J, Chuang M-H, et al. The bHLH142 transcription factor coordinates with TDR1 to modulate the expression of EAT1 and regulate pollen development in rice. The Plant Cell. 2014;26(6):2486–504. doi: 10.1105/tpc.114.126292 24894043 PMC4114947

[148] Luo J, Liu H, Zhou T, Gu B, Huang X, Shangguan Y, et al. An-1 encodes a basic helix-loop-helix protein that regulates awn development, grain size, and grain number in rice. The Plant Cell. 2013;25(9):3360–76. doi: 10.1105/tpc.113.113589 24076974 PMC3809537

[149] Wei K, Chen H. Comparative functional genomics analysis of bHLH gene family in rice, maize and wheat. BMC plant biology. 2018;18(1):1–21. 30497403 10.1186/s12870-018-1529-5 PMC6267037

[150] Wang F, Lin R, Feng J, Qiu D, Chen W, Xu S. Wheat bHLH transcription factor gene, TabHLH060, enhances susceptibility of transgenic Arabidopsis thaliana to Pseudomonas syringae. Physiological and molecular plant pathology. 2015;90:123–30.

[151] Song S, Qi T, Fan M, Zhang X, Gao H, Huang H, et al. The bHLH subgroup IIId factors negatively regulate jasmonate-mediated plant defense and development. PLoS genetics. 2013;9(7):e1003653. doi: 10.1371/journal.pgen.1003653 23935516 PMC3723532

[152] Schmiesing A, Emonet A, Gouhier-Darimont C, Reymond P. Arabidopsis MYC transcription factors are the target of hormonal salicylic acid/jasmonic acid cross talk in response to Pieris brassicae egg extract. Plant Physiology. 2016;170(4):2432–43. doi: 10.1104/pp.16.00031 26884488 PMC4825139

[153] Gao Q-M, Venugopal S, Navarre D, Kachroo A. Low oleic acid-derived repression of jasmonic acid-inducible defense responses requires the WRKY50 and WRKY51 proteins. Plant physiology. 2011;155(1):464–76. doi: 10.1104/pp.110.166876 21030507 PMC3075765

[154] Kachroo A, Lapchyk L, Fukushige H, Hildebrand D, Klessig D, Kachroo P. Plastidial fatty acid signaling modulates salicylic acid–and jasmonic acid–mediated defense pathways in the Arabidopsis ssi2 mutant. The Plant Cell. 2003;15(12):2952–65. doi: 10.1105/tpc.017301 14615603 PMC282837

[155] Kachroo P, Kachroo A, Lapchyk L, Hildebrand D, Klessig DF. Restoration of defective cross talk in ssi2 mutants: role of salicylic acid, jasmonic acid, and fatty acids in SSI2-mediated signaling. Molecular plant-microbe interactions. 2003;16(11):1022–9. doi: 10.1094/MPMI.2003.16.11.1022 14601670

[156] Pieterse CM, Van Wees SC, Van Pelt JA, Knoester M, Laan R, Gerrits H, et al. A novel signaling pathway controlling induced systemic resistance in Arabidopsis. The Plant Cell. 1998;10(9):1571–80. doi: 10.1105/tpc.10.9.1571 9724702 PMC144073

[157] Kim HS, Delaney TP. Over‐expression of TGA5, which encodes a bZIP transcription factor that interacts with NIM1/NPR1, confers SAR‐independent resistance in Arabidopsis thaliana to Peronospora parasitica. The Plant Journal. 2002;32(2):151–63. doi: 10.1046/j.1365-313x.2001.01411.x 12383081

[158] Kim HS, Delaney TP. Arabidopsis SON1 is an F-box protein that regulates a novel induced defense response independent of both salicylic acid and systemic acquired resistance. The Plant Cell. 2002;14(7):1469–82. doi: 10.1105/tpc.001867 12119368 PMC150700

[159] van Loon LC, Rep M, Pieterse CM. Significance of inducible defense-related proteins in infected plants. Annual review of phytopathology. 2006;44:135–62. doi: 10.1146/annurev.phyto.44.070505.143425 16602946

[160] Mitsuhara I, Iwai T, Seo S, Yanagawa Y, Kawahigasi H, Hirose S, et al. Characteristic expression of twelve rice PR1 family genes in response to pathogen infection, wounding, and defense-related signal compounds (121/180). Molecular Genetics and Genomics. 2008;279(4):415–27. doi: 10.1007/s00438-008-0322-9 18247056 PMC2270915

[161] Zhang HL, Jiang K, Jiang ZL, Lou H, Meng XJ, editors. Significance of PR-1 proteins in Infected Plants. Advanced Materials Research; 2014: Trans Tech Publ.

[162] Sherif S, Paliyath G, Jayasankar S. Molecular characterization of peach PR genes and their induction kinetics in response to bacterial infection and signaling molecules. Plant cell reports. 2012;31(4):697–711. doi: 10.1007/s00299-011-1188-6 22101723

[163] Tang Y, Liu Q, Liu Y, Zhang L, Ding W. Overexpression of NtPR-Q up-regulates multiple defense-related genes in Nicotiana tabacum and enhances plant resistance to Ralstonia solanacearum. Frontiers in Plant Science. 2017;8:1963. doi: 10.3389/fpls.2017.01963 29201034 PMC5696355

[164] Frick EM, Strader LC. Roles for IBA-derived auxin in plant development. Journal of Experimental Botany. 2018;69(2):169–77. doi: 10.1093/jxb/erx298 28992091 PMC5853464

[165] Tang G. Stress defense in rice: how jasmonates enhance resistance to osmotic stress: KIT-Bibliothek; 2018.

[166] Avanci N, Luche D, Goldman G, Goldman M. Jasmonates are phytohormones with multiple functions, including plant defense and reproduction. Genet Mol Res. 2010;9(1):484–505. doi: 10.4238/vol9-1gmr754 20391333

[167] Mei C, Qi M, Sheng G, Yang Y. Inducible overexpression of a rice allene oxide synthase gene increases the endogenous jasmonic acid level, PR gene expression, and host resistance to fungal infection. Molecular plant-microbe interactions. 2006;19(10):1127–37. doi: 10.1094/MPMI-19-1127 17022177

[168] Turner JG, Ellis C, Devoto A. The jasmonate signal pathway. The Plant Cell. 2002;14(suppl 1):S153–S64. doi: 10.1105/tpc.000679 12045275 PMC151253

[169] Wasternack C, Hause B. Jasmonates: biosynthesis, perception, signal transduction and action in plant stress response, growth and development. An update to the 2007 review in Annals of Botany. Annals of botany. 2013;111(6):1021–58. doi: 10.1093/aob/mct067 23558912 PMC3662512

[170] ZHILONG W. Genetic dissection of the jasmonate pathway in arabidopsis. 2006.

[171] Sharon M, Freeman S, Sneh B. Assessment of resistance pathways induced in Arabidopsis thaliana by hypovirulent Rhizoctonia spp. isolates. Phytopathology. 2011;101(7):828–38. doi: 10.1094/PHYTO-09-10-0247 21385012

[172] Glauser G, Dubugnon L, Mousavi SA, Rudaz S, Wolfender J-L, Farmer EE. Velocity estimates for signal propagation leading to systemic jasmonic acid accumulation in wounded Arabidopsis. Journal of Biological Chemistry. 2009;284(50):34506–13. doi: 10.1074/jbc.M109.061432 19846562 PMC2787311

[173] Zoeller M, Stingl N, Krischke M, Fekete A, Waller F, Berger S, et al. Lipid profiling of the Arabidopsis hypersensitive response reveals specific lipid peroxidation and fragmentation processes: biogenesis of pimelic and azelaic acid. Plant Physiology. 2012;160(1):365–78. doi: 10.1104/pp.112.202846 22822212 PMC3440211

[174] Berger S, Bell E, Mullet JE. Two methyl jasmonate-insensitive mutants show altered expression of AtVsp in response to methyl jasmonate and wounding. Plant Physiology. 1996;111(2):525–31. doi: 10.1104/pp.111.2.525 12226307 PMC157863

[175] Kachroo P, Shanklin J, Shah J, Whittle EJ, Klessig DF. A fatty acid desaturase modulates the activation of defense signaling pathways in plants. Proceedings of the National Academy of Sciences. 2001;98(16):9448–53. doi: 10.1073/pnas.151258398 11481500 PMC55441

[176] Goyal RK, Fatima T, Topuz M, Bernadec A, Sicher R, Handa AK, et al. Pathogenesis-related protein 1b1 (PR1b1) is a major tomato fruit protein responsive to chilling temperature and upregulated in high polyamine transgenic genotypes. Frontiers in plant science. 2016;7:901. doi: 10.3389/fpls.2016.00901 27446131 PMC4916175

[177] AbuQamar S, Chen X, Dhawan R, Bluhm B, Salmeron J, Lam S, et al. Expression profiling and mutant analysis reveals complex regulatory networks involved in Arabidopsis response to Botrytis infection. The Plant Journal. 2006;48(1):28–44. doi: 10.1111/j.1365-313X.2006.02849.x 16925600

[178] Jensen AB, Raventos D, Mundy J. Fusion genetic analysis of jasmonate‐signalling mutants in Arabidopsis. The Plant Journal. 2002;29(5):595–606. doi: 10.1046/j.0960-7412.2001.01241.x 11874572

