# Supplementary figures and images for: Integrative systems biology analysis of barley transcriptome ─ hormonal signaling against biotic stress
Source: PLoS One. 2023 Apr 27;18(4):e0281470. doi: 10.1371/journal.pone.0281470 (PMC10138258; doi:10.1371/journal.pone.0281470)

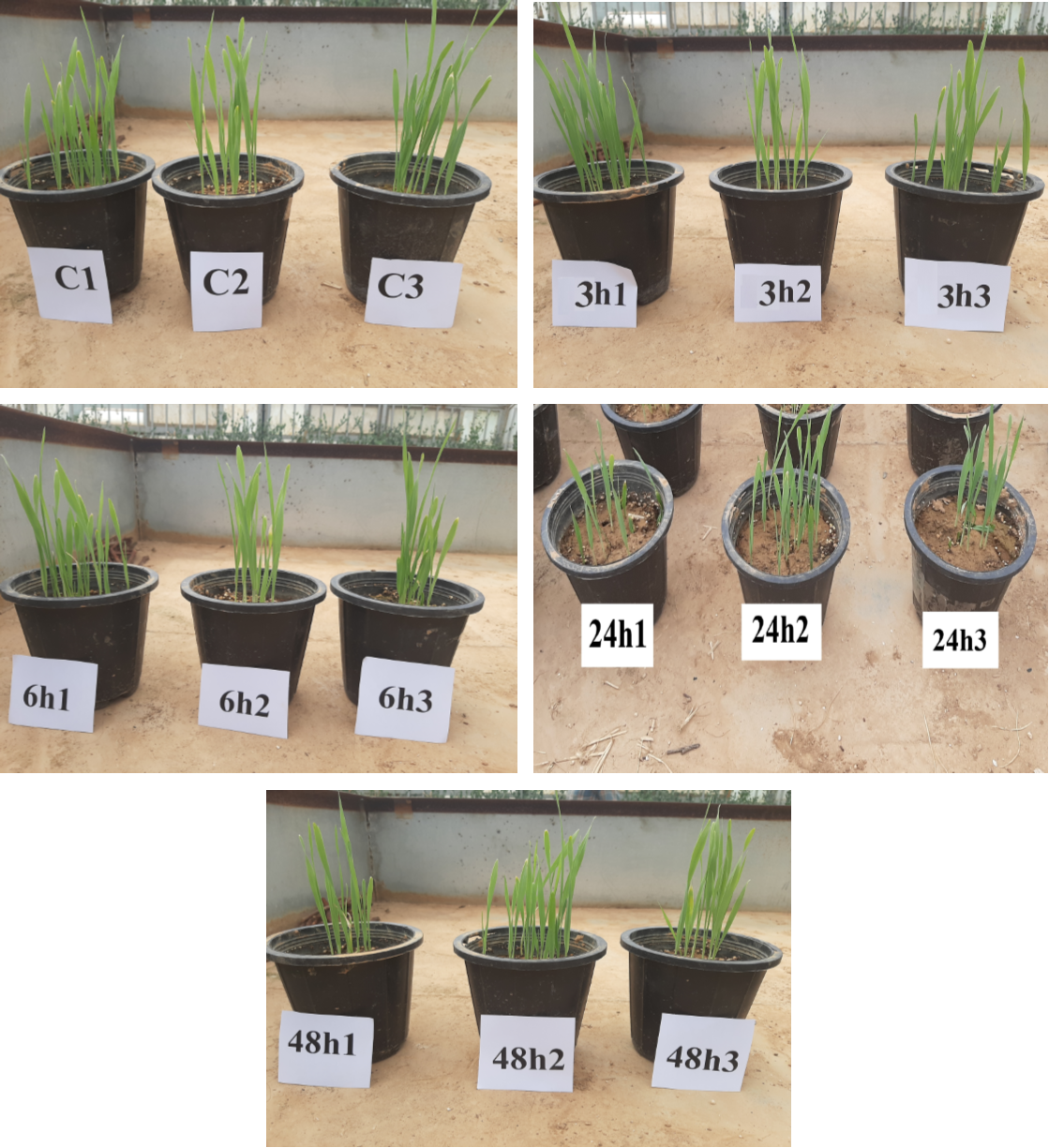

Supplement: S1 Fig — The seedlings were treated with 100 μM MeJA (plus 0.1% Tween-20) in 0.1% ethanol. In addition, the controls were sprayed and watered with 0.1% Tween-20 in 0.1% ethanol. The leaf samples were collected at 3, 6, 24, and 48 hours after treatment. (TIF) [file pone.0281470.s001.tif]

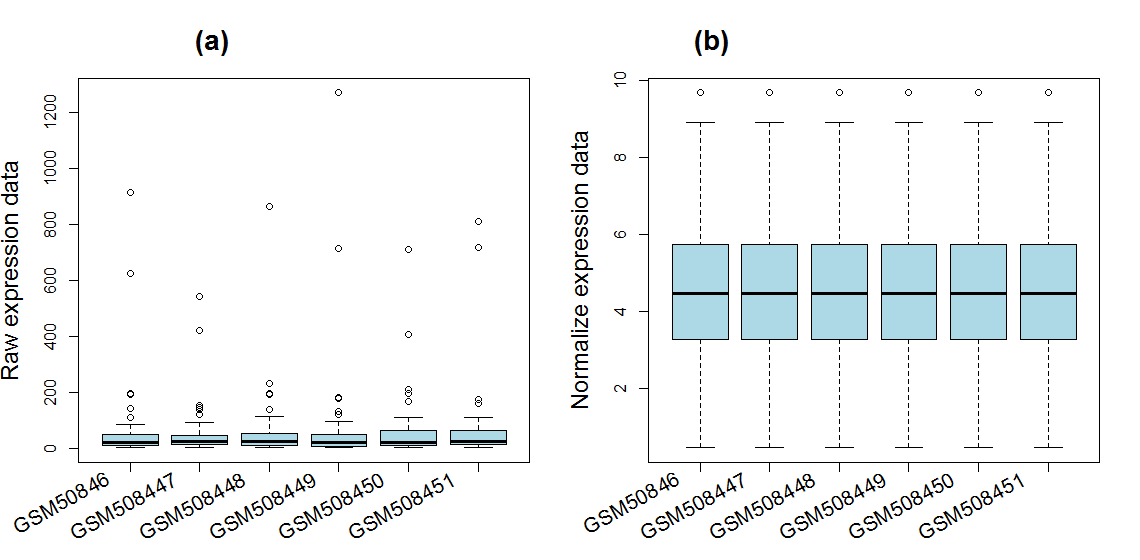

Supplement: S2 Fig — Reducing heterogeneity among samples of studies for direct merging meta-analysis. (a) The E-GEOD-33398 study plot box related to biotic stresses in Affymetrix platform with 3 control samples and 3 treatment samples, was drawn in the pre-normalization stage. (b) The E-GEOD-33398 study plot box after normalization, where all comparisons that are not significant or are not equal to the change threshold are converted to a log 2 value to remove a possible error. This method ensured that weak expression fluctuations were more likely to be real biological signals than measurement errors or errors not corrected by RMA normalization. The biological errors and batch effects have been corrected. After preprocessing, the black lines of box plot are almost on the same straight line, indicating a high level of normalization. The horizontal axis stands for control and treatment different samples, while the vertical axis represents expression value. The black line in the box represents the expression median for each sample. (TIF) [file pone.0281470.s002.tif]

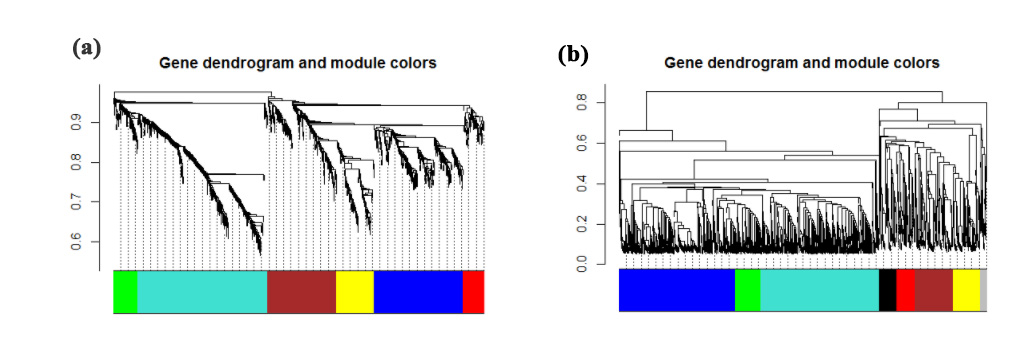

Supplement: S3 Fig — (a) Shows a hierarchical cluster tree of the biotic DEGs. The branches and color bands represent the assigned module. The tips of the branches represent genes. (b) Shows a hierarchical cluster tree of the hormonal DEGs. The branches and color bands represent the assigned module. The tips of the branches represent genes. The color bands below the dendrogram show the cluster membership (identified modules) according to tree cut methods. The X-axis refers the results of the standard, constant height cut-off method at height 0.8. The Y-axis refers to the height of the dendrogram. (TIF) [file pone.0281470.s003.tif]

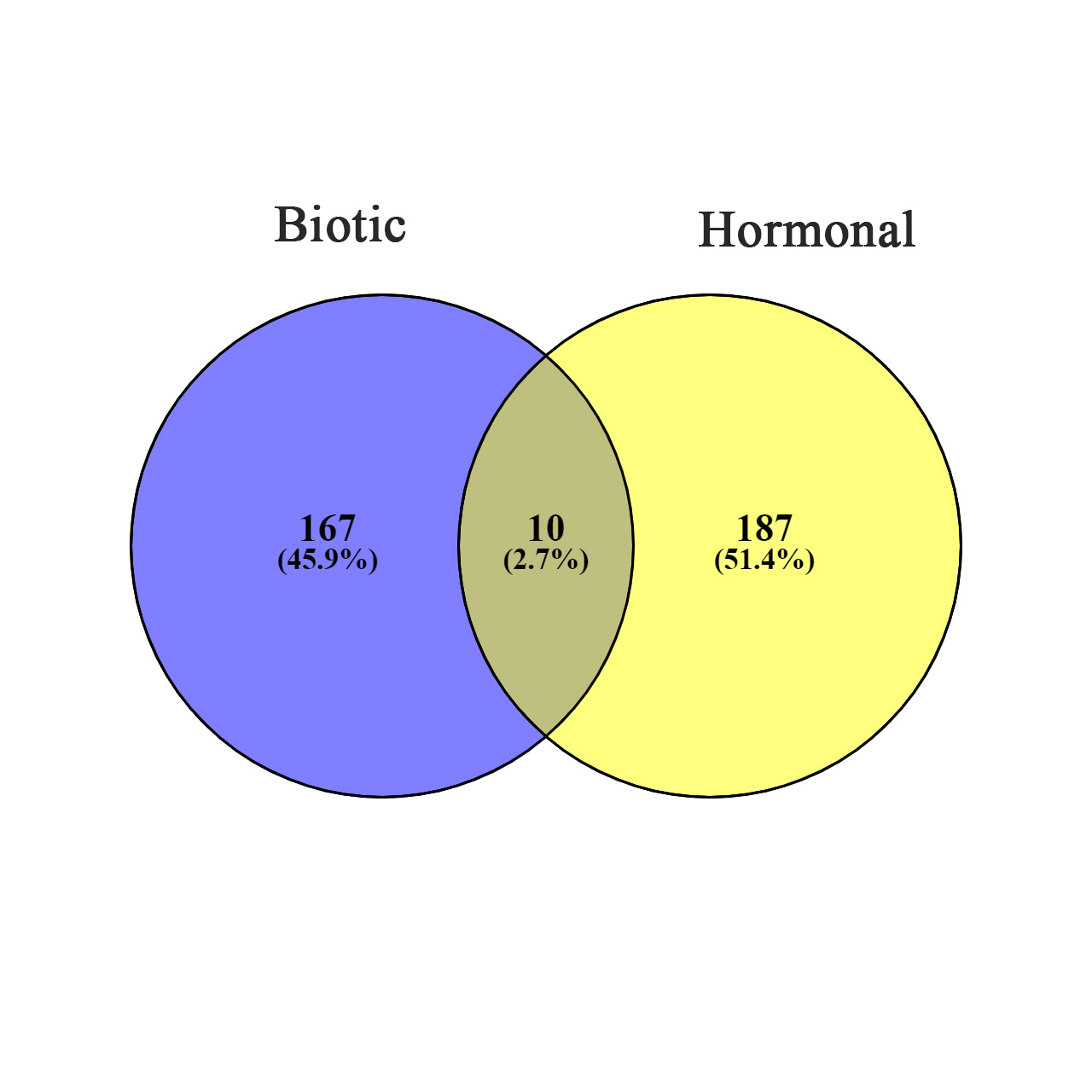

Supplement: S4 Fig — Comparison of hub genes in biotic and hormonal studies identified 10 common genes. (TIF) [file pone.0281470.s004.tif]

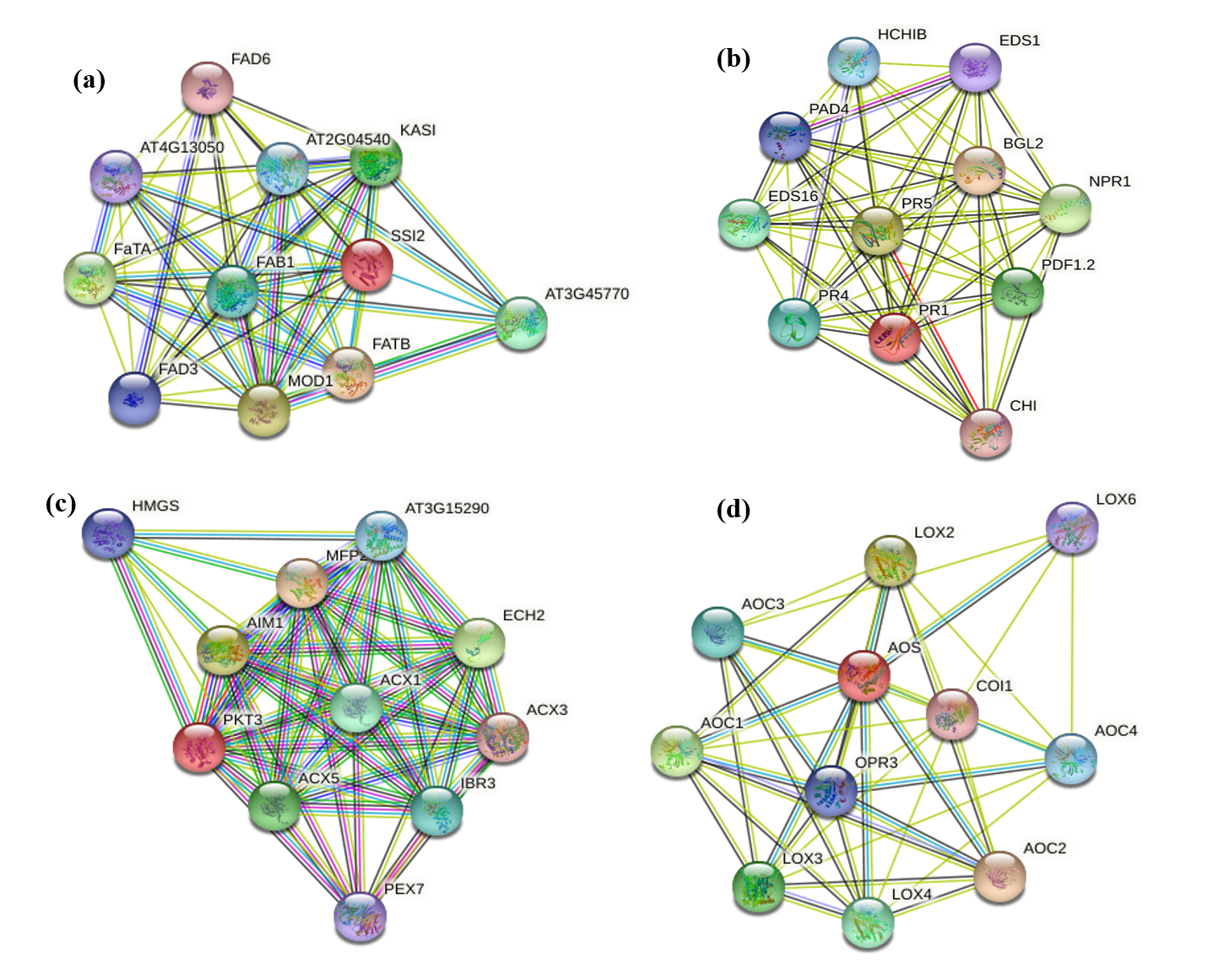

Supplement: S5 Fig — (a) The network of interactions for SSI2, (b) PR1, (c) PKT3 and (d) AOS, OPR3 and LOX2 are showed. (TIF) [file pone.0281470.s005.tif]

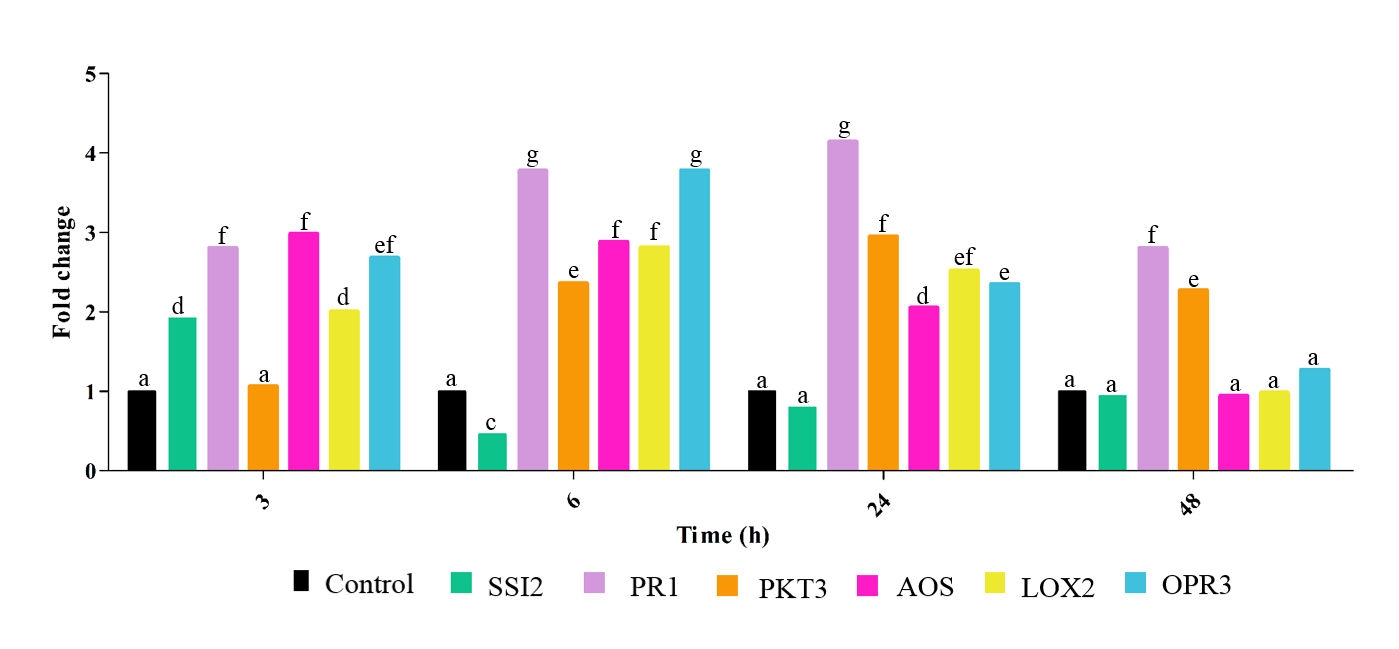

Supplement: S6 Fig — The different letters on the columns represent the significant differences given by Duncan’s multiple range statistical analysis. Vertical bars indicate ± SE of the mean (n = 3, P-value ≤ 0.05). The X-axis shows the times after MeJA treatment. As it is known, the target genes showed the highest expression at the time points of 6 h to 24 h and showed the lowest expression at the time of 48 h. (TIF) [file pone.0281470.s006.tif]

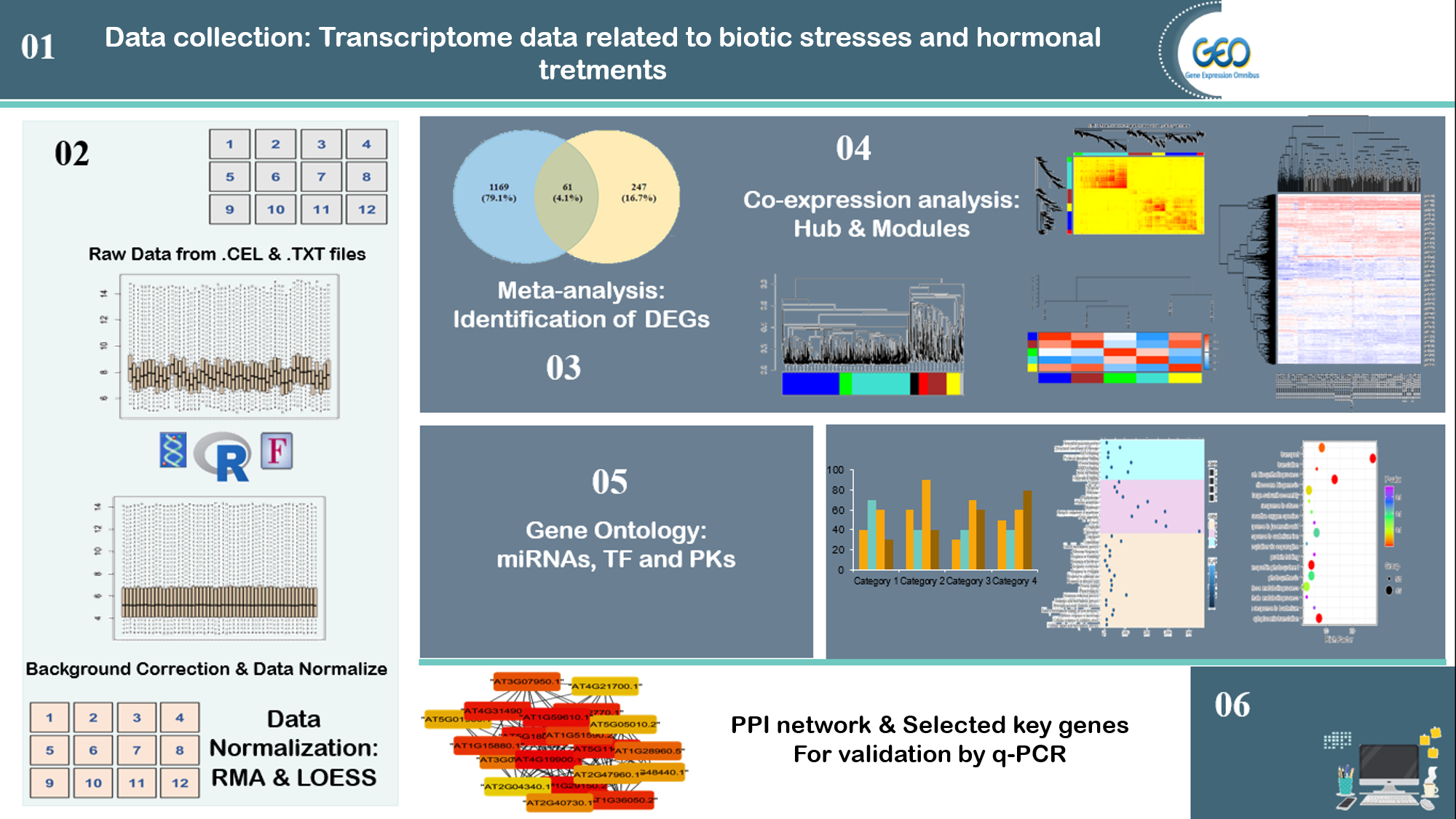

Supplement: S1 Graphical abstract — (TIF) [file pone.0281470.s017.tif]
